# Impacts of Birds vs. Invertebrate Predators on Rocky Intertidal Community Structure

**DOI:** 10.1002/ece3.71121

**Published:** 2025-03-18

**Authors:** Bruce A. Menge

**Affiliations:** ^1^ Department of Integrative Biology Oregon State University Corvallis Oregon USA

**Keywords:** bird predation, community structure, Oregon, oystercatchers, rocky intertidal, sea stars, seagulls, whelks

## Abstract

Most studies of species interactions in rocky intertidal communities focus on invertebrate predators and herbivores interacting with sessile invertebrates and macrophytes. However, shorebirds are usually a conspicuous presence on rocky shores and eat sessile and mobile invertebrate prey, often including invertebrate predators and herbivores. Inspired by classic studies of strong bird predation effects in rocky intertidal habitats in Washington state (USA) and South Africa, I tested the effects of bird and invertebrate (sea stars, whelks) predation at multiple sites, wave exposures, and zones on the central Oregon coast from spring 1996 to fall 1997. To gain insight into the effects of birds relative to the effects of invertebrate predators, I used a crossed design, with bird exclusions (present and absent) and invertebrate predator removal (present and reduced). Compared to Washington state and South Africa, birds had little effect on the abundance of sessile or mobile prey in wave‐exposed mid, wave‐exposed low, and wave‐protected mid zones at 2–4 sites. I suggest that differences between Oregon results and those in Washington and South Africa were driven by differences in bird abundance associated with whether the study site had resident colonies of shorebirds (primarily gulls, crows, and oystercatchers). That is, offshore islands often have resident breeding colonies such as in the Washington and South African studies, while sites in this study were all on the mainland where gulls were mostly transient visitors, while resident oystercatchers were usually limited to one or two pairs per site. Comparison with other marine and terrestrial experimental tests suggests that top‐down effects of birds often vary in strength, and thus, future investigations should seek to understand the factors that underlie this variation.

## Graphical abstract

I tested the relative effects of shorebird vs. invertebrate predator on prey communities on rocky shores. The experiments showed that in contrast to a similar study, birds had almost no effect on the abundances of mussels and barnacles while invertebrate predator effects were generally strong. I suggest the difference between this study and the similar study showing strong bird effects is due to habitat differences: this study was done on the mainland where birds are mostly transients while the other was done on an offshore island with resident bird colonies.

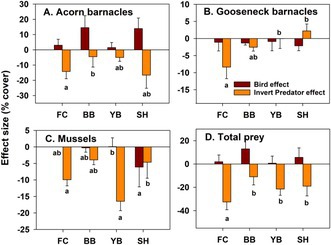

## 1 Introduction

Determining direct and indirect effects of predators on prey communities is a persistent challenge in community ecology (Wootton 1992, 1994; Menge 1995; Trussell et al. 2002; Werner and Peacor 2003; Ripple et al. 2014; Gilman 2017; Menge et al. 2024). Interest in this issue was spurred by the classic speculations of Hairston et al. (1960) who proposed that the structure of terrestrial communities was driven by strong top‐down effects of predators on herbivores that led to plants escaping herbivory, thereby dominating community structure. Later studies provided support for these ideas (e.g., Paine 1966, 1974; Estes and Palmisano 1974; Carpenter and Kitchell 1988; Schmitz 1998), and meta‐analyses explored their generality (e.g., Shurin et al. 2002).

Experimental investigations of these issues on rocky shores have advanced understanding of direct and indirect effects of predation (e.g., Wootton 1994; Menge 1995; Laska and Wootton 1998; Berlow et al. 1999; Hamilton 2000). Initially, impacts of invertebrate predators were a primary focus in rocky intertidal predator–prey investigations (e.g., Connell 1961; Paine 1966, 1974; Dayton 1971; Menge 1976; review in Menge 2000). However, birds are ubiquitous members of rocky coastlines, and many prey on intertidal invertebrates (e.g., mussels, gooseneck barnacles, whelks, limpets, chitons, crabs, and sea stars) (Hartwick 1976; Frank 1982; Irons et al. 1986; Marsh 1986a, 1986b; Wootton 1992, 1993, 1994, 1997; Hamilton 2000; Hori and Noda 2001; Hamilton and Nudds 2003; Author's personal observations). Whether or not these consumers had impacts on prey populations or communities on the US west coast, however, was unclear until the experiments of Marsh (1986a, 1986b) and Wootton (1992, 1994). On the Oregon coast, Marsh (1986a, 1986b) showed that shorebirds negatively affected the abundance of high intertidal small mussels (surfbirds, *Calidris virgata*; gulls, *Larus* spp., and black oystercatchers *Haematopus bachmani* ) and high intertidal limpets (primarily black turnstones, *Arenaria melanocephala*, black oystercatchers and gulls). On Tatoosh Island, Washington state, Wootton (1992, 1994, 1997) showed that birds negatively affected the abundance and size structure of limpets ( *Lottia digitalis* and *L. pelta* ) and reduced the abundance of gooseneck barnacles ( *Pollicipes polymerus* ). These studies also revealed important indirect effects on macrophytes (through consumption of limpets [herbivores]) and mussels (through consumption of gooseneck barnacles [space competitors]).

Inspired by these results, I conducted experiments testing separate and joint effects of birds and invertebrate predators on rocky intertidal communities along the central Oregon coast in spring 1996 to fall 1997. Prior studies in this region had shown important effects of whelks on mid intertidal zone prey, especially barnacles and mussels (Navarrete 1996; Berlow 1997, 1999) and of sea stars on low intertidal zone prey (Menge 1992; Menge et al. 1994; Navarrete and Menge 1996), and that these effects varied through time. In addition to the studies of Marsh (1986a, 1986b), my field observations of regurgitated seagull pellets at two sites on the central Oregon coast indicated that gulls preyed on a variety of invertebrates. For example, at Fogarty Creek (see below), prey included gooseneck barnacles (*Pollicipes polymerus*, 16 pellets), whelks (*Nucella* spp., 5), small mussels ( *Mytilus trossulus* , 2), and crabs (1). At the Florence (Oregon) jetty, prey included *P. polymerus* (18), *Balanus glandula* (13), *M. trossulus* (15), crabs (5), fish bones (2), and *Nucella* spp. (1) (Author's personal observations). At Strawberry Hill, I have observed clusters of gooseneck peduncles (i.e., the stalk attachment) lacking the capitulum (i.e., the part including most of the barnacle soft parts), and gulls tugging the capitulum off and swallowing it. Given the predominance of gooseneck barnacles in these observations, I was interested in the generality of the effects observed by Wootton (1992, 1994, 1997), i.e., if they also occurred along the Oregon coast. I was also interested in whether this predation had community effects. For example, Wootton (1994) showed that in the absence of gull predation, mussel displacement of gooseneck barnacles via space competition (e.g., Paine and Levin 1981) was slowed.

In this study, I asked four questions. First, does shorebird predation have effects on prey communities like those seen on Tatoosh Island? Second, given earlier experiments showing strong invertebrate predator effects, I was curious about potential interactions between shorebird and invertebrate predation. I asked: Do shorebird effects differ when invertebrate predators are absent from when they are present, and vice versa? Third, given the among‐site and among‐zone differences in community structure and predation strength observed in prior studies (Marsh 1986a, 1986b; Menge 1992; Menge et al. 1994), I wanted to determine how shorebird–invertebrate predator effects varied among sites, between zones, and at different wave exposures. I asked: Do shorebird and invertebrate predator effects differ by intertidal zone, between wave‐exposed and wave‐protected benches, or among sites along the central Oregon coast? Finally, I wanted to compare shorebird abundances at Oregon sites to those documented by Wootton (1997).

## 2 Methods

### 2.1 Natural History

Rocky intertidal communities along the North America west coast harbor a diverse set of predators. As noted by Marsh (1986a, 1986b) and Wootton (1992, 1994, 1997), common bird species feeding on intertidal taxa include sea gulls (glaucous‐winged gulls *Larus glaucescens* and western gulls *L. occidentalis* ), oystercatchers ( *H. bachmani* ), American crows (*Corvus brachyrhyncos*), surfbirds (*Calidris* [*Aphriza*] *virgata*), and black turnstones ( *Arenaria melanocephala* ). Relevant invertebrate predators include the sea star *Pisaster ochraceus* , the six‐armed sea star *Leptasterias* spp., whelks (*Nucella ostrina*, *N. canaliculata*, *Lirabuccinum dirum* [formerly *Searlesia dira* ]), and crabs (*Cancer productus*, *Glebocarcinus* [formerly *Cancer*] *oregonensis*) (e.g., Dayton 1971; Palmer 1980; Menge et al. 1994; Navarrete 1996; Navarrete and Menge 1996; Berlow 1997; Noda 1999). Of these, gulls, oystercatchers, the sea stars, and the whelks are by far the most abundant and/or most consistently present (Marsh 1986a, 1986b; Wootton 1992, 1994, 1007; Author's personal observations). Surfbirds (https://birdsoftheworld.org/bow/species/surfbi/cur/introduction) and black turnstones (https://birdsoftheworld.org/bow/species/blktur/cur/introduction) are present only during the fall, winter, and early spring months. They typically breed in the Arctic in spring and early summer and return to Oregon in late summer, leaving again in April. Gulls, crows, and oystercatchers are year‐round residents.

The primary prey of these predators is limpets (*Lottia* spp.), barnacles (*Balanus glandula, Semibalanus cariosus, Pollicipes polymerus*) and mussels ( *Mytilus trossulus* and *M. californianus* ) (above references). Prior research has shown that whelks and the small sea star *Leptasterias* spp. prey primarily on acorn barnacles and small mussels (Dayton 1971; Menge 1972; Menge et al. 1994; Navarrete 1996; Navarrete and Menge 1996; Berlow 1997; Noda 1999; Wootton 2002; Novak et al. 2017). The sea star *Pisaster ochraceus* preys heavily on the smaller acorn barnacles and mussels, as well as the larger mussel *M. californianus* and the gooseneck barnacle *P. polymerus* .

### 2.2 Study Sites

**FIGURE 1:**
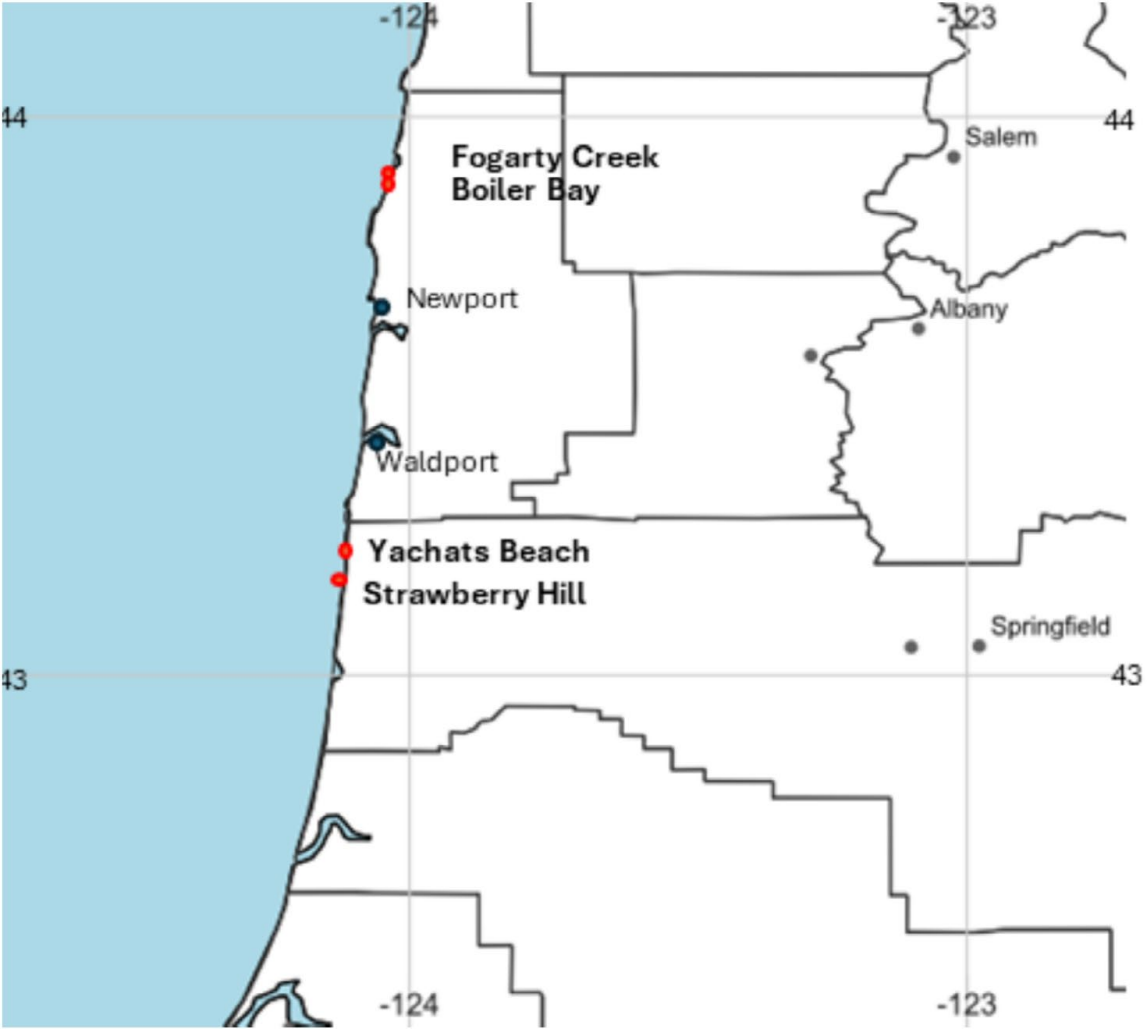
Map showing study areas. Fogarty Creek and Boiler Bay are on Cape Foulweather (northern sites) and Yachats Beach and Strawberry Hill are on Cape Perpetua (southern sites). SimpleMappr (https://www.simplemappr.net/) was used to make the map.

Experiments were conducted at two to four sites located on two capes (regions) depending on zone and exposure (Figure 1), where zone refers to shore levels (mid = middle zone, low = low zone) and exposure refers to variation in wave forces. Exposed mid (hereafter EM) experiments were done at Fogarty Creek (44.84° N−124.6° W), Boiler Bay (44.83° N−124.06° W), Yachats Beach (44.32° N−124.11° W), and Strawberry Hill (44.25° N−124.11° W) (hereafter FC, BB, YB, and SH, respectively). Exposed low (EL) intertidal and protected mid (PM) experiments were done at BB and SH. All sites have been described in detail in previous studies (e.g., see Menge et al. 1994, 1997, 2004, 2015; Gravem et al. 2024), but briefly are broad benches with typical zonation patterns, with the high zone dominated by barnacles and fucoid algae, the mid zone dominated by mussels, and the low zone dominated by macrophytes (kelp, turf‐forming red algae, surfgrass). Anemones ( *Anthopleura xanthogrammica* ) are also common in the low zone. In the EL zone, the relative dominance of these functional groups and the invertebrate predators varies along the coast, with macrophytes being more luxuriant and sessile and mobile invertebrates being less abundant at Cape Foulweather sites (FC and BB), and macrophytes less prominent and sessile and mobile invertebrates being more abundant at Cape Perpetua sites (YB and SH).

### 2.3 Experimental Design

**FIGURE 2:**
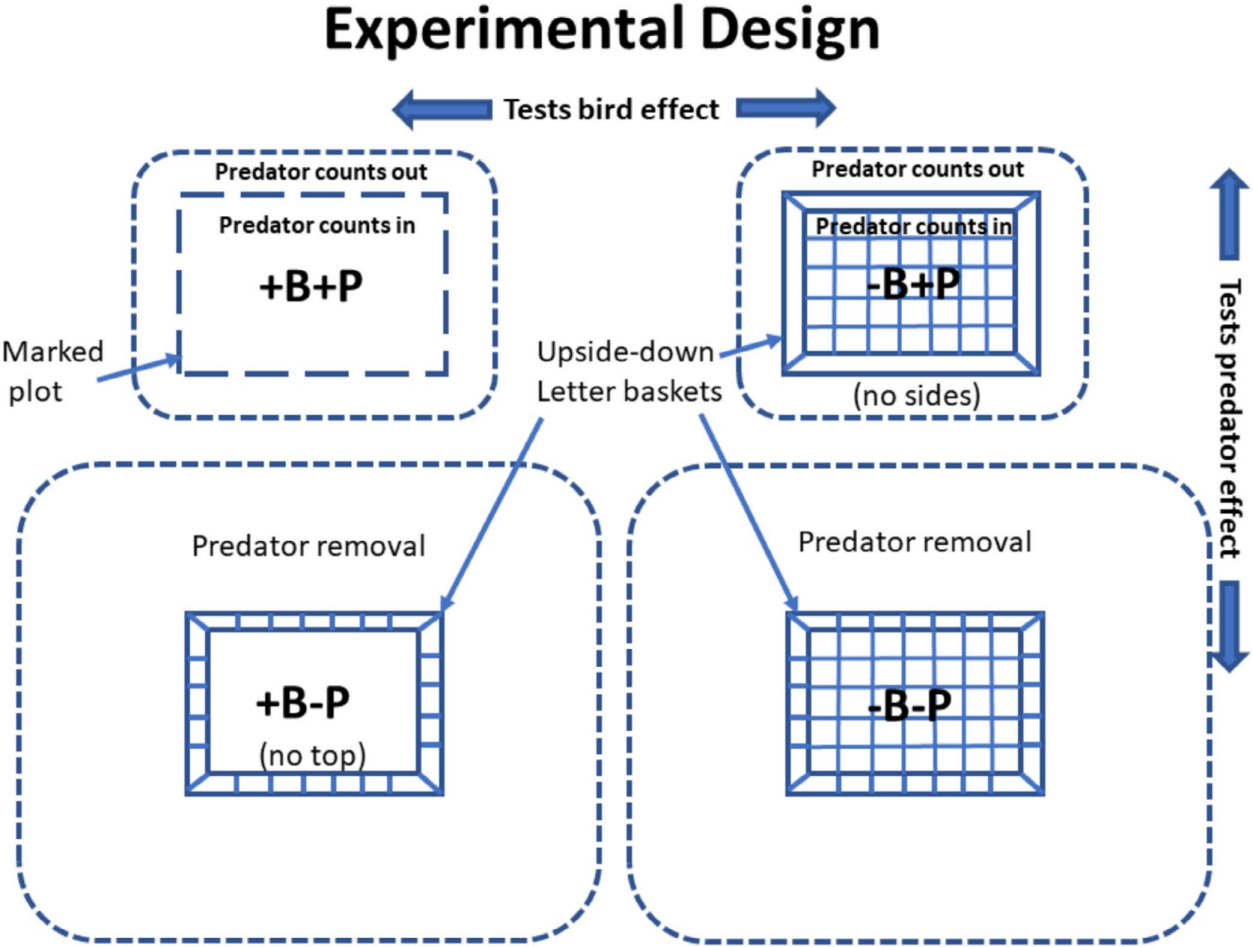
Experimental design. Treatments are birds (B) and invertebrate predators (P) present (+) or absent (−). Dotted lines show the bands around each plot in which invertebrate predators were counted (top panels) or counted and removed (bottom panels). The wider band around the plots in the bottom panels shows the area cleared of prey (mussels, barnacles, mobile species) and shelter (macrophytes).

To ensure that my results were comparable to Wootton's (1994), I used upside‐down vinyl‐covered wire letter baskets fastened to the rock as the basic bird exclusion device (Figures S1–S3). Like Wootton (1994), basket tops were removed to allow bird access, and sides were removed to ease access by larger predators (e.g., *P. ochraceus* ) while intact baskets excluded birds, and plots marked with lag screws on the corners served as controls. Because the baskets did not prevent entry by whelks and small sea stars (and determined large sea stars), invertebrate predators were manipulated manually. To slow reinvasion of cages or experimental plots by invertebrate predators, all prey and macrophytes were cleared from a band of approximately 30 cm width around invertebrate predator exclusions (see Figure S3). The idea was that whelks and small sea stars would be deterred by these preyless and shelterless bands and tend to remain in more prey‐ and shelter‐rich spaces away from the cage or plot. Since all community components (sessile invertebrates, algae) recruit to the rock from the plankton, this treatment should have had no effect on abundances colonizing the cleared plots. Thus, the orthogonal experimental design had four treatments, marked plots (+Birds and +Invertebrate Predators, hereafter coded as +B+P), sideless cages (−B+P), topless cages with a cleared band around them (+B−P), and intact cages with a cleared band around them (−B−P) (Figure 2). Because Wootton (1994) had tested possible cage artifacts (e.g., by installing cages and marked plots on vertical surfaces where gulls could not access the plots, testing if the cage rim deterred whelk entry) and found no effects, we did not establish similar procedural controls.

The exposed mid intertidal habitat was most like that used by Wootton (1994) on Tatoosh Island, so replicated experiments (*n* = 5) were established in this zone at four sites (FC, BB, YB and SH). Each replicate included the four treatments listed above (e.g., see Figure S3), and each treatment was separated from others by about 1 m. Replicates were spread across about 20–30 m of shore at each site. To assess how results varied with wave exposure and tidal zone, identically designed and replicated experiments were initiated at PM benches and EL zones at BB and SH (personnel and time constraints prevented setting up these combinations at FC and YB).

Experiments were monitored every low tide series (i.e., about every other week) from April to September and every ~1–2 months from October to March when wave conditions limited access. The reduced frequency of monitoring in winter likely had little effect on results since low tides are at night from October to March, so bird foraging intertidally is greatly reduced. Further, invertebrate predator foraging is also reduced due to heightened wave action and cooler temperatures, both of which inhibit feeding (e.g., Menge 1978a, 1978b; Menge et al. 1994, 2002; Sanford 1999). To establish a uniform starting point for all treatments, experiments were initiated by clearing plots and installing markers and cages in June 1996 and terminated by removing hardware in September 1997 (16 months). On each visit, the percent cover of sessile organisms was estimated in each plot, all invertebrate predators within cages, and in a 30 cm wide zone around each plot were counted, and in −P plots were removed. Cages and hardware were replaced or repaired as needed.

### 2.4 Bird Surveys

Bird abundance estimates were obtained from the literature (Liebezeit et al. 2020; Wootton 1997), from a NOAA database (https://catalog.data.gov/dataset/oregon‐coast‐nwrc‐comprehensive‐seabird‐colony‐catalog‐database/resource/ecece07d‐dd2a‐4444‐bfa8‐76a8a5ddca87), and from counts made in a recent study in 2018–19 (detailed results to be reported elsewhere). Wootton (1997) presented both population census numbers and the mean number of birds/100 m of shoreline taken monthly over a 5‐year period (1985–1990). I extracted estimates of his monthly samples (during the breeding season, ~April to August) from his Figures 1 and 2 using a ruler to line up data points with the *y*‐axis scale.

Although I did not conduct counts during the 1996–97 experiment, NOAA's Seabird Colony Catalog included historic counts of oystercatchers and gulls at multiple mainland and offshore island sites ranging back to the 1980s and 1990s. NOAA estimates of abundance were based on direct counts of all birds at the specific site but did not include information on site area or shoreline length. NOAA data included breeding bird colonies on two offshore sites, as well as the number of breeding birds censused on mainland sites. I extracted relevant data to examine temporal changes and overall average abundances of these two taxa. Crows were not included in this Catalog.

The recent survey method involved a research team of ~2–8 individuals that scanned the study site for ~15 min before descending from the cliff overlooking the site to conduct field studies. Observers used binoculars to identify and count the number of each species present at the site. One individual served as data recorder. In some cases, additional observations were made from the overlook before departing the site as the tide advanced. All but one observation was made at or shortly after dawn. Records were kept of the presence of humans (14 occurrences in 47 surveys), dogs (one in 47 surveys), and harbor seals (3 in 47 surveys). Separate counts were made for birds that were flying, swimming, on land above the site, and on the rocks. Note was made if the bird observed was foraging, but in most cases, the specific prey could not be identified but likely included mussels, gooseneck barnacles, limpets, and whelks. Oregon data were presented as both average total number observed per site and number/100 m of shoreline. Estimates of shoreline length observed during our counts were obtained using the ruler on Google Earth.

### 2.5 Data Analysis

Data were analyzed using JMP v16.1.0 (SAS Institute Inc., 2021–2022), PRIMER 7, and PERMANOVA+ for PRIMER (Anderson and Gorley 2008). Data were natural log‐transformed (ln (*x* + 1)) for JMP analyses and square root‐transformed for PRIMER analyses.

Community‐level analysis, including acorn and gooseneck barnacles, mussels, anemones, macrophytes, and bare space, and pairwise tests were conducted using PERMANOVA+ (Anderson et al. 2008). PERMANOVA+ model factors included site, bird treatment, invertebrate predator treatment (all fixed), sample time, and their interactions. Separate tests were done for wave exposure and zone (EM, EL, and PM) combinations. PERMANOVA estimates of variance components were used to examine the relative importance of each factor and interaction. I used PERMDISP to test the homogeneity of multivariate dispersions.

Because community‐wide analyses can mask possible effects of interactions among community components, I followed these analyses by testing the effects of site, birds, and invertebrate predators on the prey taxa (acorn and gooseneck barnacles, mussels) using univariate analyses of variance. Effect sizes of birds and invertebrate predators on acorn barnacles, gooseneck barnacles, and mussels were quantified as the per replicate difference in percent cover between +B and −B treatments (i.e., −B minus +B) and +P and −P treatments (i.e., −P minus +P), respectively, and tested using analysis of variance. Each difference was multiplied by −1 so negative (positive) effects would be negative (positive) numbers.

Nonmetric multidimensional scaling (nMDS) was used to plot mean positions of community centroids for each analysis to visualize differences among treatments by site. Non‐transformed data were plotted in figures, and analyses used transformed data. In univariate comparisons, pairwise tests used linear contrasts and Tukey HSD tests to determine ranks of sites for each significant factor or interaction. Variance explained in univariate analyses was estimated using eta‐squared for each factor and adjusted *R* ^2^ for the full model.

## 3 Results

### 3.1 Bird Surveys

**FIGURE 3:**
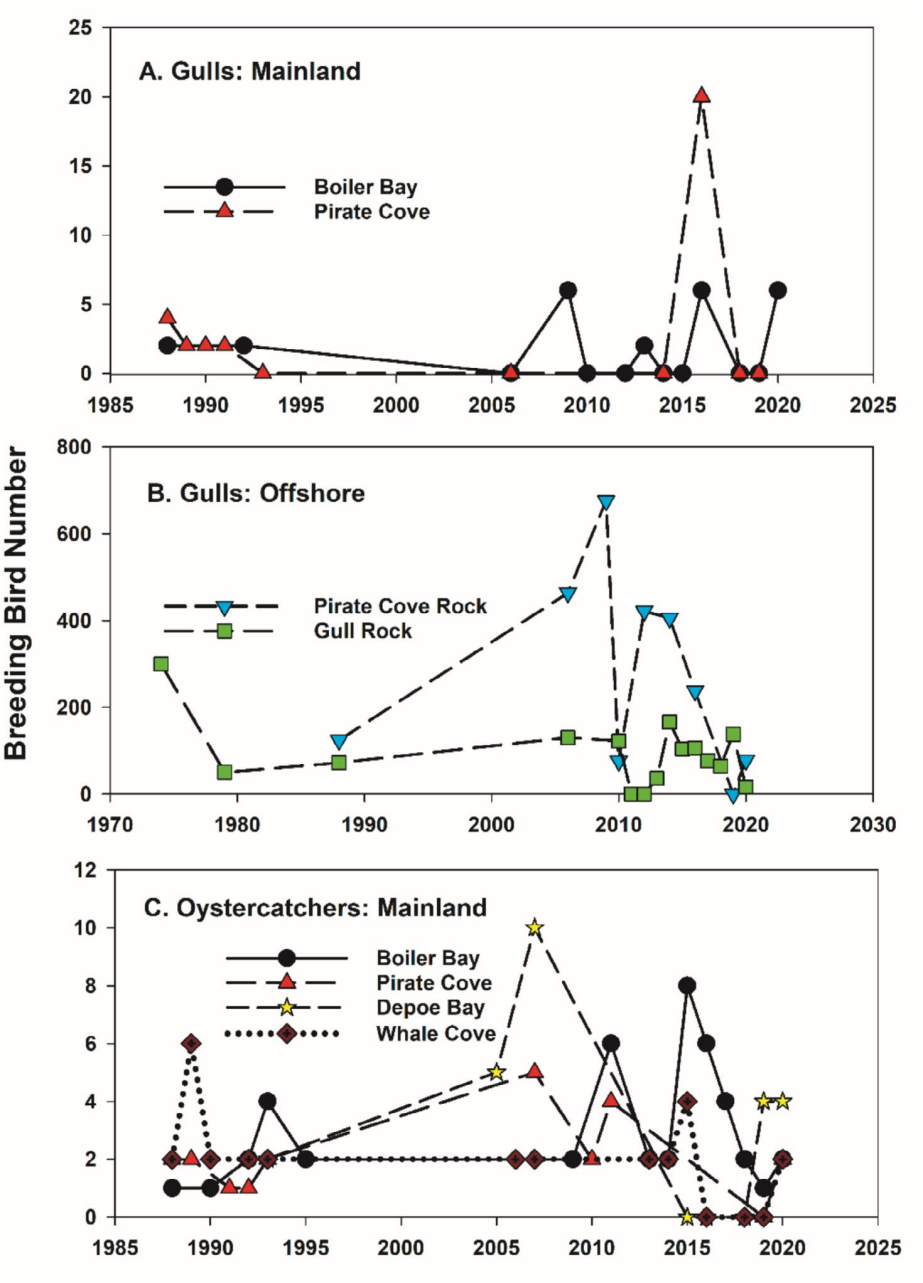
Abundance of gulls ( *Larus glaucescens* and *L. occidentalis* ) and oystercatchers ( *Haematopus bachmani* ) at mainland (A and C) and offshore island sites (B) along the central Oregon coast.

NOAA counts of breeding bird abundance, typically done in spring and summer, were done sporadically but extended as far back as the 1970s and 1980s for the sites relevant to this study (Figure 3, Table 1). For gulls, counts were made at mainland sites in the Cape Foulweather area (i.e., where FC and BB are located) and at two offshore rocks. While abundances fluctuated through time, the data indicate that the number of breeding gulls were generally low on the mainland (Figure 3A, Table 1, mean [± 1 standard error] numbers ranged from 0.33 ± 0.33 to 4.9 ± 0.6 in the Cape Foulweather region) and substantially higher on the offshore rocks (Figure 3B, Table 1, mean numbers were 56.5 ± 14.8 and 276 ± 76 at the two offshore rocks in this region).

**TABLE 1:** Abundance of breeding oystercatchers ( *Haematopus bachmani* ) and sea gulls ( *Larus glaucescens* and *L. occidentalis* ) on mainland and offshore sites along the central Oregon coast.

Site	Latitude	Longitude	Habitat	Taxon	Mean ± 1SE	Sample size
Boiler Bay	44.8297	−124.0591	Mainland	*Haematopus bachmani*	3 ± 0.55	15
Pirate Cove Rock	44.8196	−124.0669	Offshore	*Haematopus bachmani*	2.1 ± 0.46	10
Gull Rock	44.7518	−124.0748	Offshore	*Haematopus bachmani*	2.92 ± 0.23	26
Devil's Punch Bowl	44.7458	−124.0656	Mainland	*Haematopus bachmani*	2.67 ± 0.47	9
Cape Perpetua	44.2867	−124.1154	Mainland	*Haematopus bachmani*	1.67 ± 0.33	3
Captain Cook Point	44.2747	−124.1139	Mainland	*Haematopus bachmani*	1 ± 1	2
Strawberry Hill	44.2571	−124.1121	Mainland	*Haematopus bachmani*	1 ± 1	2
Gwynn Knoll	44.2465	−124.1152	Mainland	*Haematopus bachmani*	4	1
Boiler Bay	44.8297	−124.0591	Mainland	*Larus* spp.	1.85 ± 0.7	14
Pirate Cove	44.8186	−124.0650	Mainland	*Larus* spp.	3 ± 1.94	10
Pirate Cove Rock	44.8196	−124.0669	Offshore	*Larus* spp.	276 ± 76	15
Depoe Bay North	44.8154	−124.1643	Mainland	*Larus* spp.	1.43 ± 0.57	7
Depoe Bay Bridge	44.8098	−124.0620	Mainland	*Larus* spp.	4.9 ± 0.6	18
Whale Cove	44.7884	−124.0675	Mainland	*Larus* spp.	0.33 ± 0.33	6
Gull Rock	44.7518	−124.0748	Offshore	*Larus* spp.	56.4 ± 14.75	30
Cape Perpetua	44.2867	−124.1154	Mainland	*Larus* spp.	1 ± 1	2
Strawberry Hill	44.2571	−124.1121	Mainland	*Larus* spp.	1 ± 1	2

*Note:* Data are from the NOAA Seabird Colony Catalog (https://catalog.data.gov/dataset/oregon‐coast‐nwrc‐comprehensive‐seabird‐colony‐catalog‐database/resource/ecece07d‐dd2a‐4444‐bfa8‐76a8a5ddca87).

For oystercatchers, numbers varied temporally but were usually < 10 breeding birds per site both at mainland and offshore sites (Figure 3C, Table 1). The surveys of Liebezeit et al. (2020) are consistent with these data and suggest that along the Oregon coast, oystercatcher numbers have remained stable, with densities in recent surveys being similar to those conducted in 2006.

Two caveats are obvious in these data. First, few counts were made in the Yachats/Cape Perpetua area (where YB and SH are located). Second, due to budget limitations (R. Suryan, personal communication) no data were collected at the relevant sites between about 1993 and 2002. At some latitudes, observations were recorded from 1994 to 1997 as “present” but not at my sites. However, at three sites in the Newport, OR region, counts made in 1994 to 1997 were 40, 38, 42, and 38; 12, 14, 10, and 10; and 14, 18, 22, and 16, perhaps suggesting consistency at the regional scale.

**FIGURE 4:**
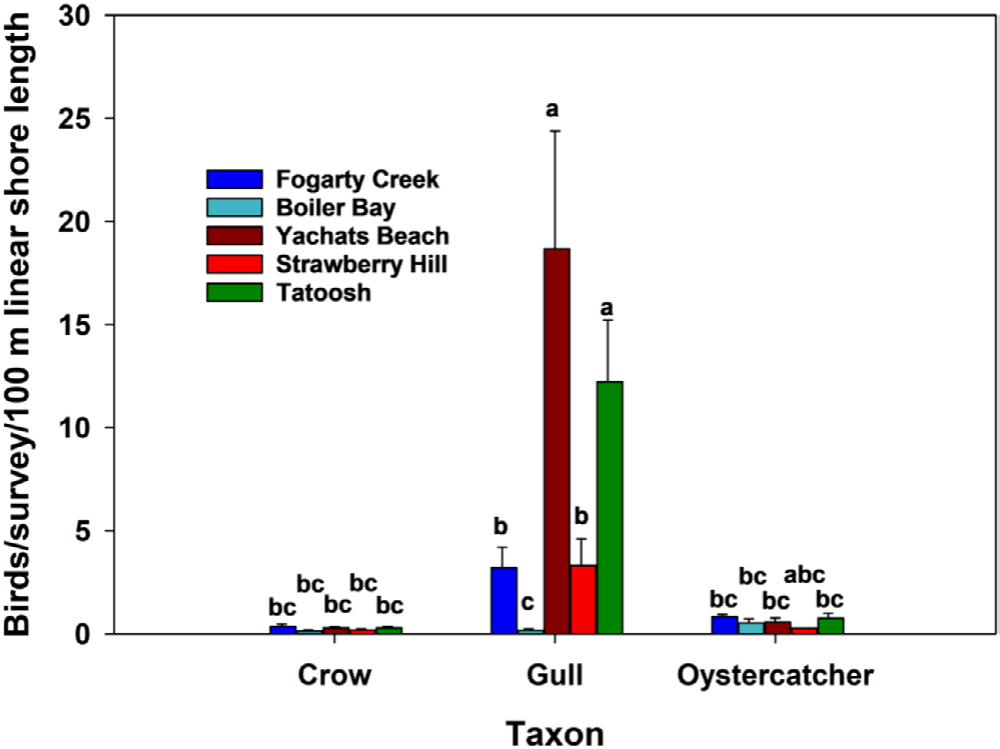
Abundances of crows, gulls, and oystercatchers at the four study sites and on Tatoosh Island. Data were collected in 2018–2019 for the Oregon sites, and in 1985–1990 by Wootton (1997) at Tatoosh, and are means and standard errors. Bars sharing the same letters are not different at *p* < 0.05.

In the 2018–2019 surveys, gulls were by far the most abundant bird taxon, confirming my long‐held subjective assessments (Figure 4, Table 2). Gulls were most abundant at YB (~80/survey) and similarly abundant at FC, BB, and SH (~10–23/survey). Crows ranged from 1.3 to 2.3/survey, and oystercatchers ranged from 2 to 2.8/survey, and abundances of these two taxa did not differ among sites (Table 2).

**TABLE 2:** One‐way analysis of variance testing differences in counts of crows, gulls, and oystercatchers taken in visual surveys at four study sites.

Species	df	*F*	*p*	Adj. *R* ^2^	Tukey HSD
A. Total across categories					
Crows (*Corvus brachyrhyncos*)	3, 20	0.92	0.45	−0.01	FC = BB = YB = SH
Gulls ( *Larus glaucescens* , *L. occidentalis* )	3, 49	7.15	**0.0004**	0.262	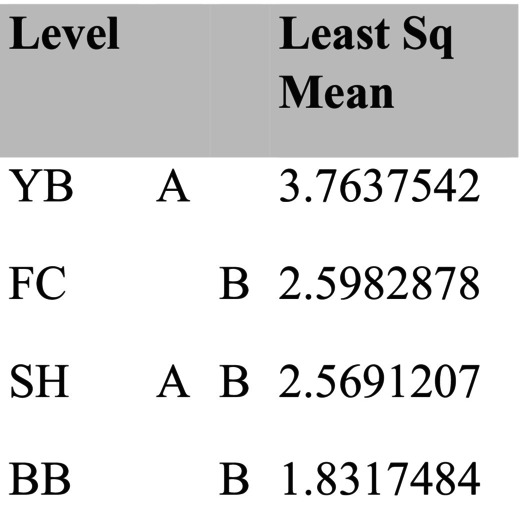
Black Oystercatcher ( *Haematopus bachmani* )	3, 20	0.16	0.92	−0.123	FC = BB = YB = SH
B. Number in intertidal					
Crows (*Corvus brachyrhyncos*)	3, 20	0.69	0.57	−0.042	FC = BB = YB = SH
Gulls (*Larus glaucescens, L. occidentalis*)	3, 49	14.16	**< 0.0001**	0.432	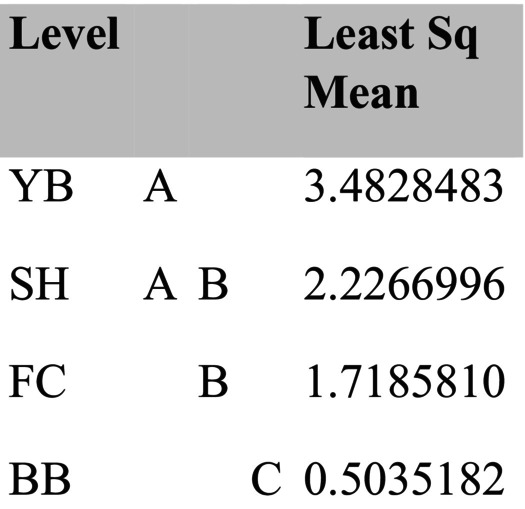
Black Oystercatcher ( *Haematopus bachmani* )	3, 20	0.04	0.99	−0.143	FC = BB = YB = SH

*Note:* Analyses of counts totalled across subcounts taken of birds that were flying over the site, on nearby land, in the supratidal (splash zone), in the intertidal, and swimming nearby are shown in A, and for intertidal only in B. Count data were ln‐transformed (ln (*x* + 1)). At Fogarty Creek, Boiler Bay, Yachats Beach, and Strawberry Hill, respectively, gulls were observed on 16/16, 14/14, 13/13, and 10/10 surveys; crows on 9/16, 7/16, 5/13, and 3/10 surveys; and oystercatchers on 13/16, 4/14, 6/13, and 1/10 surveys. The significance level is *p* < 0.05.

Wootton's (1997) censuses indicated that the breeding gull colony on Tatoosh Island was large, averaging 2280 ± 150 (*n* = 26) birds. Oystercatcher and crow numbers were far less, 12.8 ± 1 and 15.1 ± 1.1, respectively. Interestingly, when standardized to 100 m of shoreline, gull numbers on Tatoosh were similar to those at one Oregon site, Yachats Beach (Figure 4), but, as expected given the huge size of the breeding colony, much higher than all other Oregon sites. Crow and oystercatcher densities at Tatoosh were similar to those at the Oregon sites.

### 3.2 Experiments

In general, bird effects on prey taxa were weak relative to those of invertebrate predators. Further, at the prey community level, bird effects were observed only at one site, SH; no bird effects were found at the level of individual prey taxa.

**FIGURE 5:**
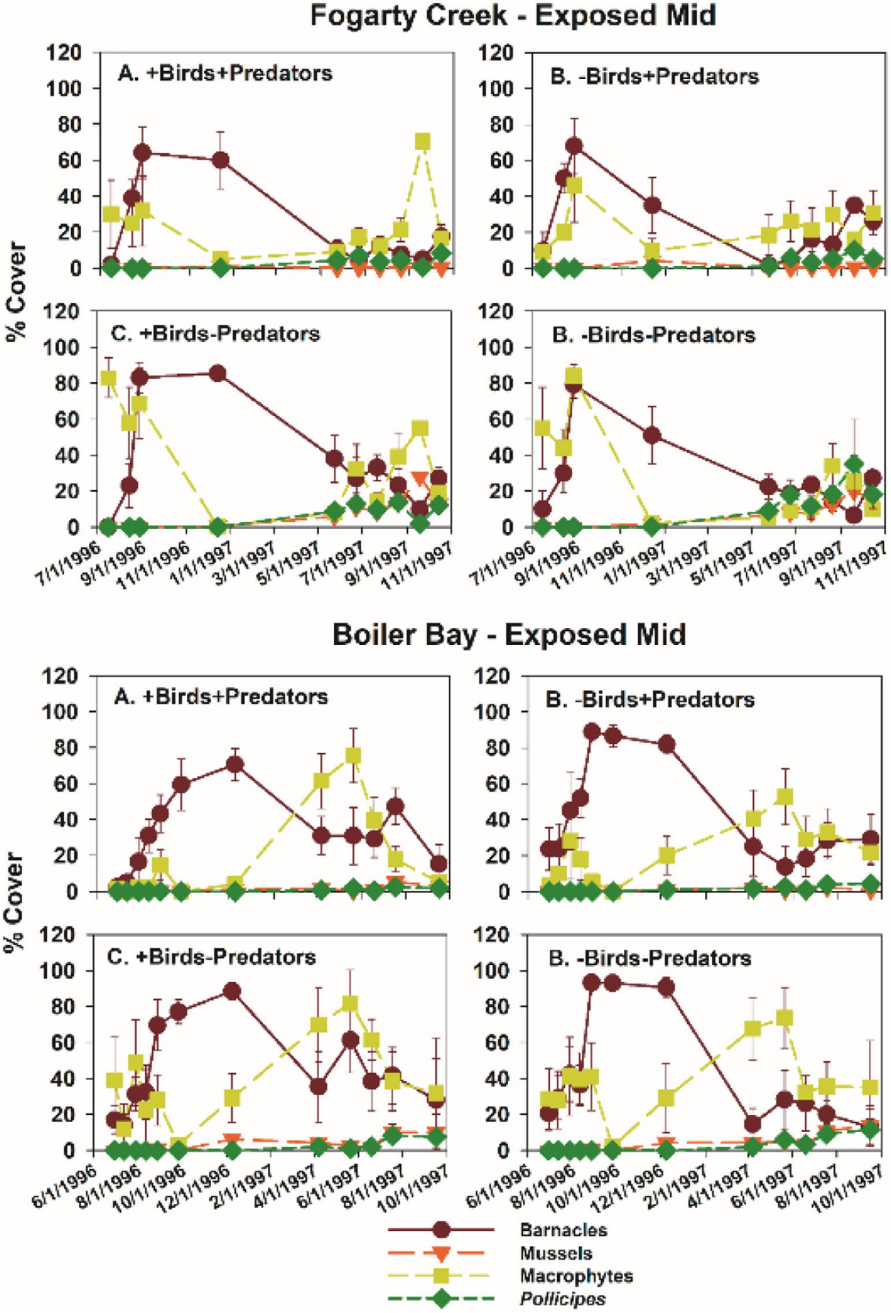
Time series of changes in the four major taxa responding to experimental manipulations in each treatment in Fogarty Creek Exposed Mid and Boiler Bay Exposed Mid zones. Data points are means and 1 standard error in this and following figures.

**FIGURE 6:**
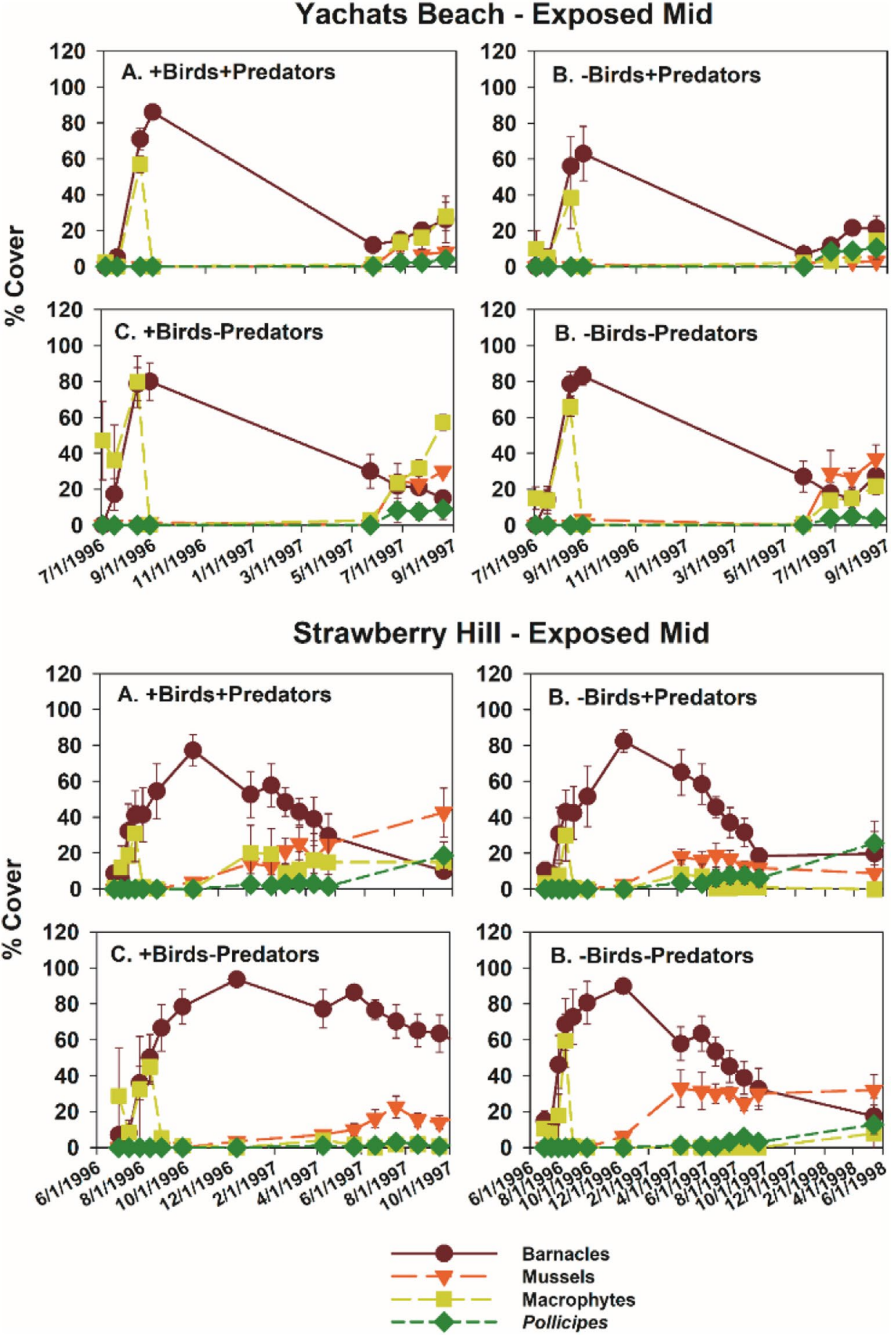
Time series of changes in the four major taxa responding to experimental manipulations in each treatment in Yachats Beach Exposed Mid and Strawberry Hill Exposed Mid zones.

Typically, during the time course of the experiments, acorn barnacles and macrophytes were early colonists. Macrophyte cover then declined during fall and winter, while acorn barnacles persisted but usually declined into the second summer (Figures 5 and 6). Colonization of gooseneck barnacles, favored as prey by birds, and larger mussels took longer than early colonists, so analyses focused on data from the second spring and summer (April through September) 1997.

#### 3.2.1 Exposed Mid

**FIGURE 7:**
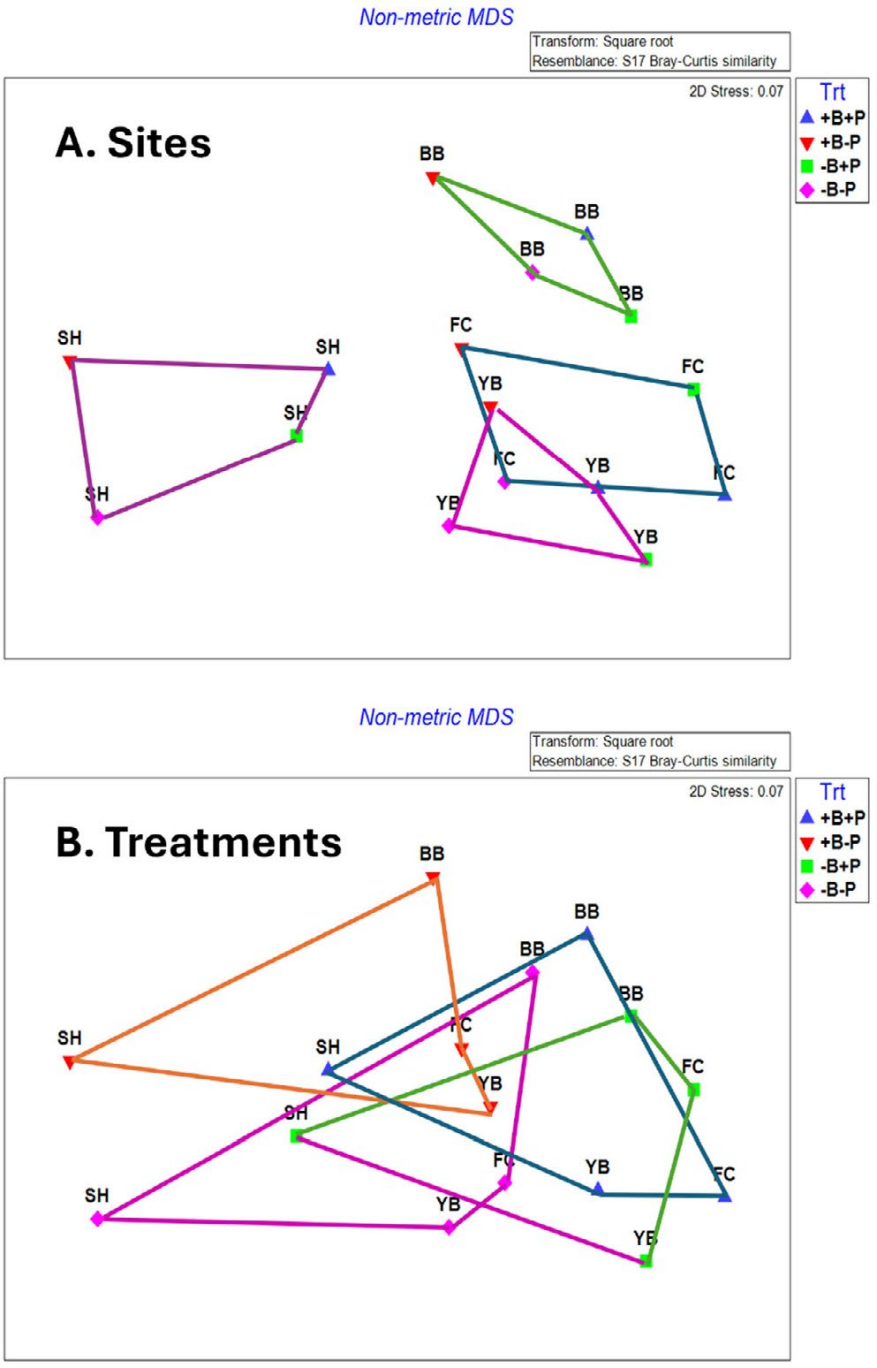
nMDS ordination plots for exposed mid zone results. (A) Site envelopes and (B) Treatment envelopes.

By summer 1997, community structure was affected by all factors, either as a main effect (invertebrate predators, sample time) or through interactions with other effects (site, birds) (Table 3). However, the relative influences of each main effect varied considerably. Summing across the components of variation (main effects plus each term that included the main effect), site had the strongest overall effect (33.05), followed by time (26.08), invertebrate predators (7.72), and birds (−0.66) (Table 3). Pairwise tests show that invertebrate predators had significant effects in all but one site × bird treatment case, while birds had no effect in all but one site × invertebrate predator case (Table 4). Tests for homogeneity of multivariate dispersions indicated that the site × treatment group was not homogeneous (*p* = 0.001), an effect primarily reflecting site differences (*p* = 0.001) since treatments were homogeneous (*p* = 0.92) (Table 5, Figure 7; site results overlapped little while treatment results overlapped extensively in ordination space). These analyses included several categories of non‐prey (anemones, macrophytes, bare space), so to deepen insight into effects on prey species, I examined how acorn barnacles, gooseneck barnacles, and mussels varied by site and treatment.

**TABLE 3:** Exposed Mid intertidal zone: PERMANOVA test of effects of site, birds and invertebrate predators, and sample date (time) on community structure in field experiments.

Source	df	SS	MS	Pseudo‐*F*	*p*(perm)	Unique permutations	Components of variation
Site	3	76,146	25,382	2.10	0.079	999	8.45
Bird effect	1	1751.9	1751.9	3.39	0.055	999	2.14
Pred effect	1	21,860	21,860	39.86	**0.001**	999	8.89
Sample time	13	1.3361e+05	10,277	24.83	**0.001**	998	13.06
Site × bird effect	3	5963	1987.7	5.79	**0.004**	999	4.20
Site × predator effect	3	5595.9	1865.3	4.55	**0.003**	999	3.95
Site × sample time	26	3.1547e+0.05	12,133	29.32	**0.001**	996	24.58
Bird × pred effect	1	1114.4	1114.4	4.86	**0.026**	999	2.56
Bird effect × sample time	13	7400.3	569.25	1.38	0.129	995	2.32
Pred effect × sample time	13	8003.5	615.66	1.49	0.083	997	2.64
Site × bird × pred effect	3	2022.7	674.23	9.20	**0.002**	999	3.59
Site × bird effect × sample time	26	8918.9	343.04	0.83	0.762	999	−2.70
Site × pred effect × sample time	26	10,661	410.05	0.99	0.48	994	−0.63
Bird × pred × sample time	13	1779.7	136.9	0.33	0.99	999	−4.38
Site × bird × pred × sample time	26	1882	72.38	0.17	1.00	999	−8.39
Residual	667	2.7605e+05	413.87				23.34
Total	838	8.9684e+05					

*Note:* Sites were Fogarty Creek, Boiler Bay, Yachats Beach, and Strawberry Hill. The analysis was limited to the final months of the experiment, i.e., from April to September 1997. Community structure was the square‐root transformed abundance (percent cover) of acorn barnacles, gooseneck barnacles, mussels, anemones, macrophytes, and bare space. The analysis used a Bray–Curtis similarity resemblance matrix, with Type III (partial) sums of squares. Time was random, and other factors were fixed, and the method was permutation of residuals under a reduced model. The significance level is *p* < 0.05.

**TABLE 4:** Pair‐wise tests using PERMANOVA of effects of birds in invertebrate predator treatments and invertebrate predators in bird treatments in mid‐exposed experiments at four sites.

Site	Bird effect		Predator effect	
	+P	−P	+B	−B
Fogarty Creek	0.2	0.82	**0.001**	**0.001**
Boiler Bay	0.443	0.117	**0.027**	0.08
Yachats Beach	0.122	0.694	**0.012**	**0.004**
Strawberry Hill	0.247	**0.001**	**0.001**	**0.001**

*Note:* Data in the resemblance matrix were Bray–Curtis similarities based on square root transformed percent cover data. The analysis used Type III (partial) sums of squares with fixed effects summing to zero for mixed terms with 999 permutations of residuals under a reduced model. The analysis was based on 1997 data obtained in approximately monthly samples from April through September. The values are probabilities based on *t*‐tests. The significance level is *p* < 0.05.

**TABLE 5:** PERMDISP tests for homogeneity of multivariate dispersions of community data in experiments.

Group	No. of groups	No. of samples	Test of deviations	Resemblance	*F*	df	*p*(perm)
A. Exposed mid							
Site × treatment	16	389	Centroid	Bray–Curtis Similarity	6.70	15, 373	**0.001**
Site	4	389	Centroid	Bray–Curtis Similarity	18.61	3, 385	**0.001**
Treatment	4	389	Centroid	Bray–Curtis Similarity	0.18	3, 385	0.92
B. Exposed low							
Site × treatment	8	174	Centroid	Bray–Curtis Similarity	4.31	7, 166	**0.01**
Site	2	174	Centroid	Bray–Curtis Similarity	19.39	1, 172	**0.001**
Treatment	4	174	Centroid	Bray–Curtis Similarity	1.02	3, 170	0.57
C. Protected mid							
Site × treatment	8	219	Centroid	Bray–Curtis Similarity	6.21	7, 211	**0.001**
Site	2	219	Centroid	Bray–Curtis Similarity	6.66	1, 217	**0.015**
Treatment	4	219	Centroid	Bray–Curtis Similarity	10.48	3, 215	**0.001**

*Note:* Data were square root transformed. The number of permutations was 999 in all cases. The significance level is *p* < 0.05.

**FIGURE 8:**
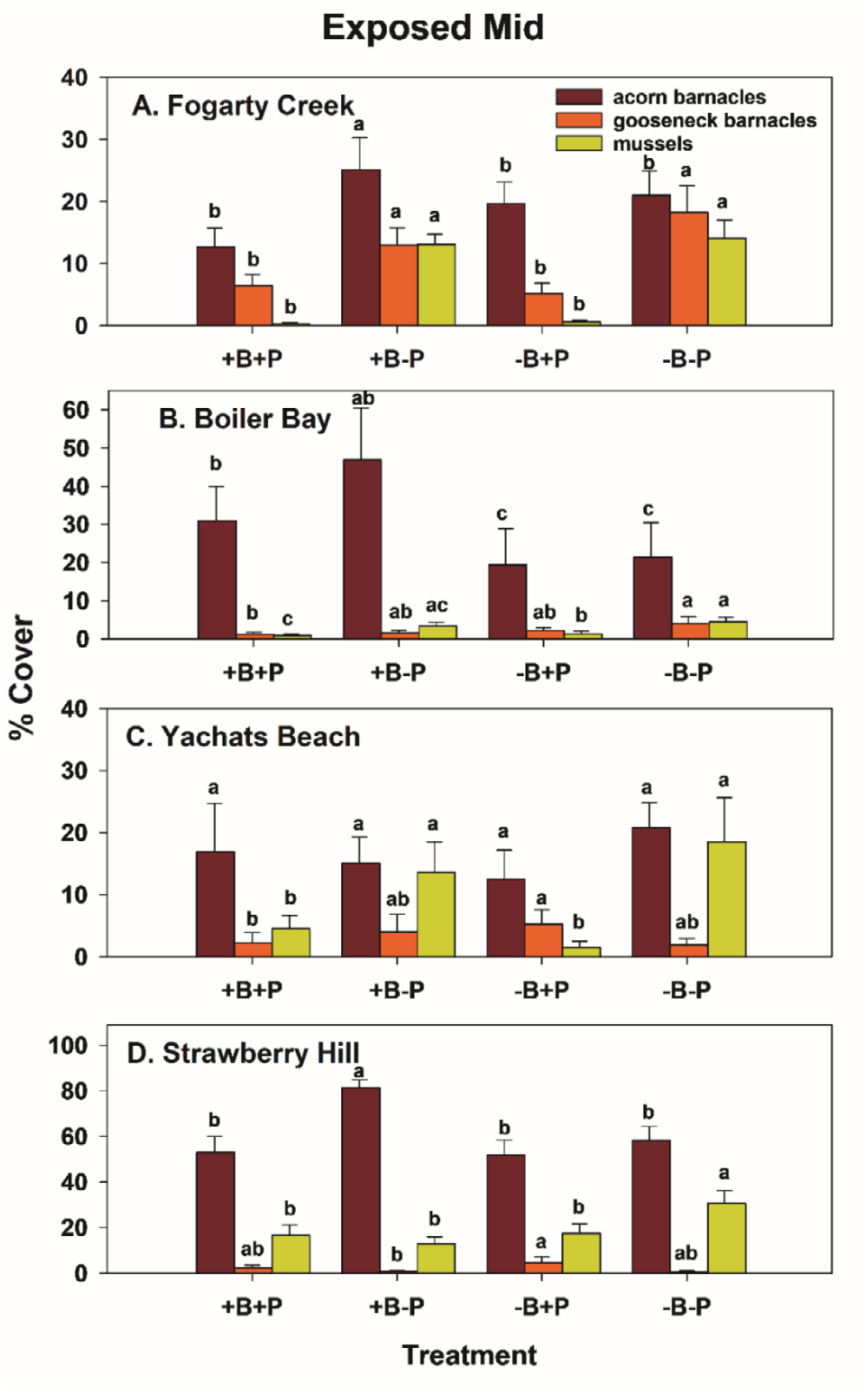
Percent cover (mean and 1 SE [standard error]) of acorn barnacles, gooseneck barnacles, and mussels in mid exposed experiments at Fogarty Creek (A), Boiler Bay (B), Yachats Beach (C) and Strawberry Hill (D). Pairwise comparisons within (but not between) taxa used least squares contrasts. Bars sharing lower‐case letters indicate no among‐treatment differences at *p* < 0.05.

Among‐site differences in sessile invertebrate prey in the EM experiments were substantial (Figure 8, Table S1). Although univariate analyses suggested bird predation affected all three taxa, either as a main effect (both barnacle types) or through site × bird interactions (mussels), pairwise comparisons (linear contrasts) suggested that bird effects on acorn barnacles were likely indirect. That is, this taxon tended to be less, not more, abundant in the absence of birds (Figure 8A,B,D) or did not vary among treatments (Figure 8C, Table S1). Similarly, bird effects on gooseneck barnacles and mussels were detected, but these effects were small, inconsistent, and likely ecologically unimportant (Figure 8, Table S1).

In contrast, relatively large invertebrate predator effects were detected for all three prey taxa (Table S1), especially for mussels and gooseneck barnacles at FC, and for mussels at YB (Figure 8). Based on the variance explained for each effect, invertebrate predator effects ranged from 2.03 (acorn barnacles) to 16 (mussels) times stronger than bird effects in the EM habitat.

**FIGURE 9:**
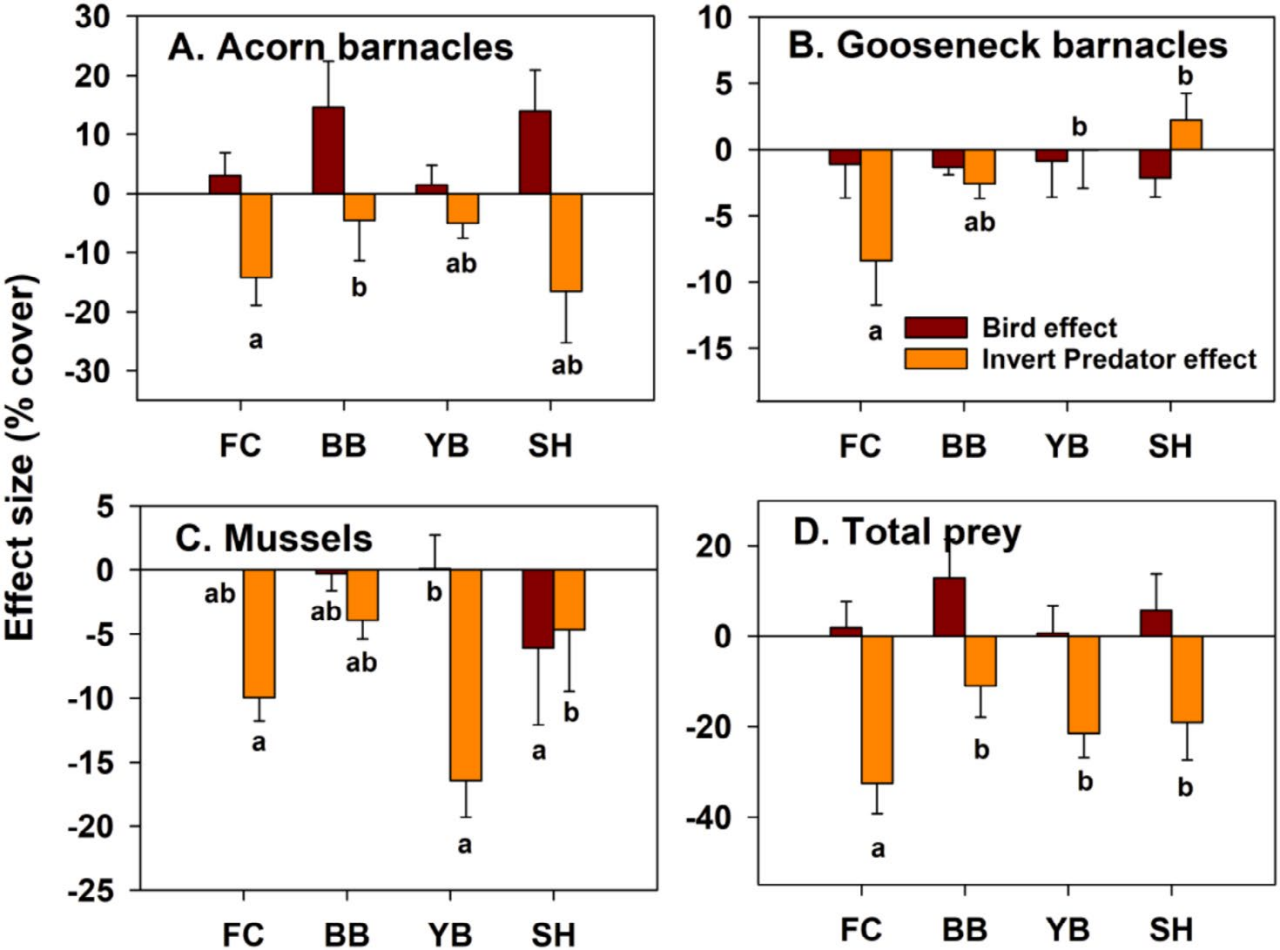
Effect size for exposed mid experiments, quantified as the per replicate difference between +B vs. −B (purple bars) and +P vs. −P treatments (orange bars), for acorn (A) and gooseneck barnacles (B), mussels (C), and the sum of these taxa (D). Bars sharing lower‐case letters indicate no among‐treatment differences at *p* < 0.05. Pairwise comparisons employed linear contrasts. Bird effects did not differ between sites so bars were not coded.

**FIGURE 10:**
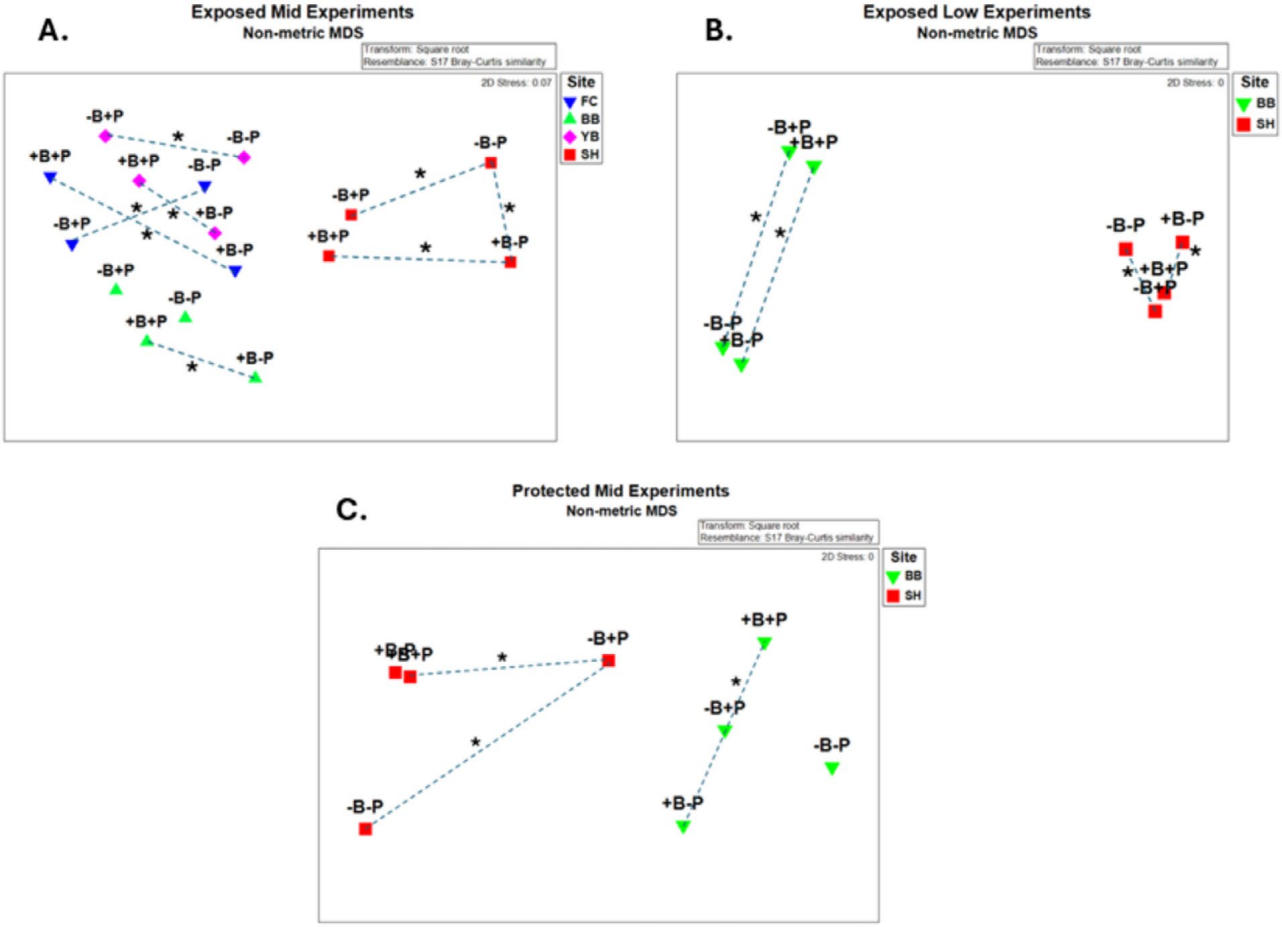
Non‐Metric Multi‐dimensional Scaling of 1997 means of each treatment at each site for the (A) exposed mid zone, (B) exposed low zone, and (C) protected mid zone experiments. Dashed lines with asterisks indicate pairs at each site that differ as determined by pair‐wise tests using PERMANOVA (see Tables 3, 6 and 8).

Effect size estimates confirmed these results and further indicated that bird effects were generally unimportant in EM experiments (Figure 9). Specifically, mean effects on prey in bird absence were smaller than those in bird presence, so effect sizes were positive (range = 1.5%–14.6% more abundant with birds present). Although negative effect sizes occurred with gooseneck barnacles and mussels at most sites, they were small (−0.9% to −2.1% for goosenecks, 0.1 to −6.1 for mussels) (Figure 9B,C). Bird effect sizes for total prey abundance were also small (0.7%–13% more abundant with birds) and reflected the positive pattern seen for acorn barnacles (Figure 9D). In contrast, invertebrate predator effect sizes were much larger and mostly negative, with the largest effects occurring with acorn barnacles and mussels (Figure 9); total prey abundance decreased by 11%–32.6%. These trends are consistent with those shown using ordinal space (nMDS) methods (Figure 10A), where +B−P and −B−P symbols are more widely separated than +B+P and −B+P symbols for each site.

**FIGURE 11:**
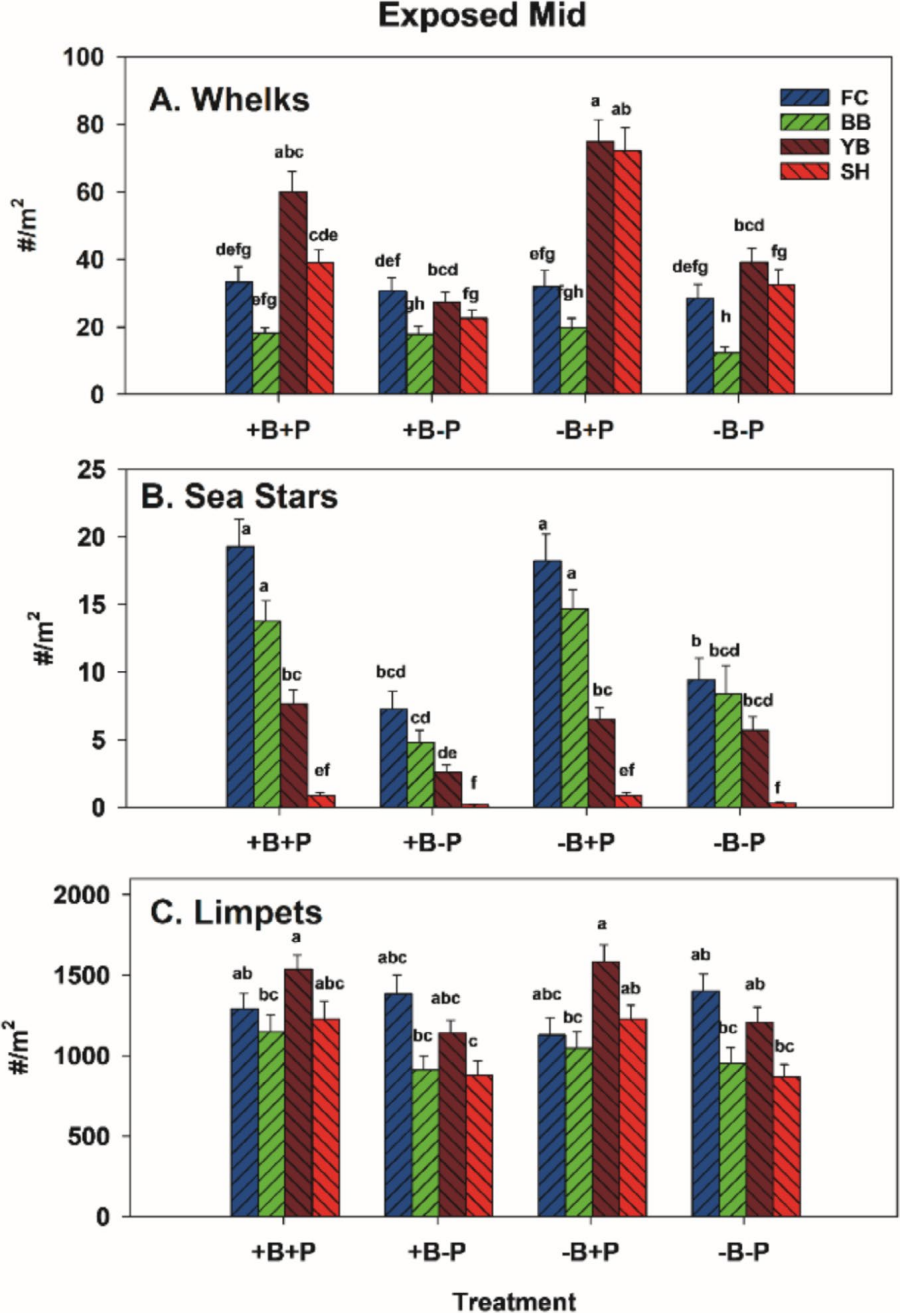
Overall mean number of whelks (A) and sea stars (B) either counted in (+P plots) or removed from (−P) plots by site. We also tracked limpet abundance (C) for comparison to possible indirect effects of manipulations on macrophytes. The lower‐case letters indicate differences among treatments and sites, with no difference at *p* < 0.05 for those bars sharing the same letter.

**FIGURE 12:**
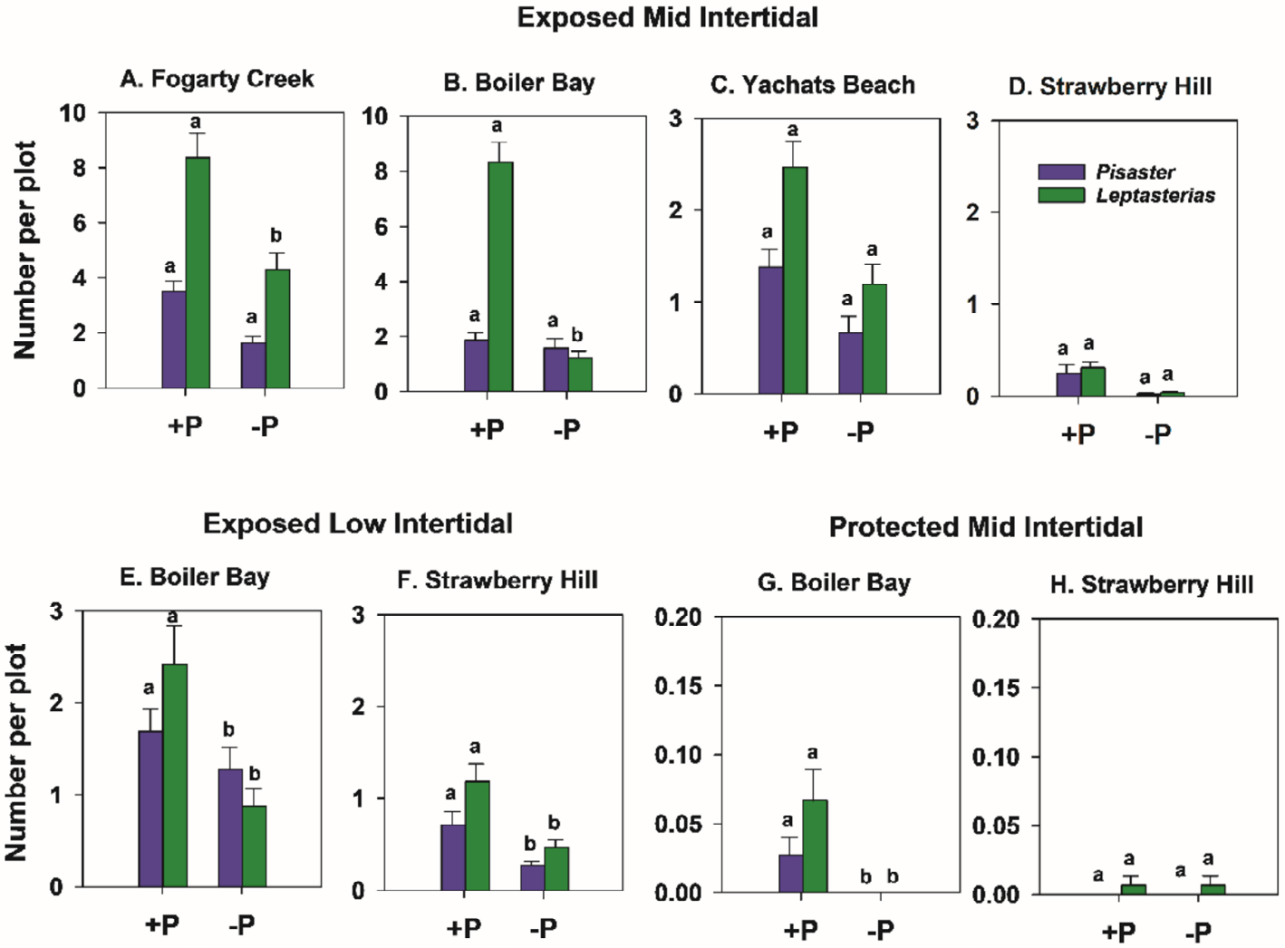
Mean densities of *Pisaster ochraceus* and *Leptasterias* sp. at each site in exposed mid (A–D), exposed low (E, F) and protected mid intertidal zones (G, H). The lower‐case letters indicate within taxon differences among treatments, with no difference at *p* < 0.05 for those bars sharing the same letter.

Effectiveness of excluding invertebrate predators in −P plots varied by site × taxon (Figure 11). Whelks were reduced at SH and YB −P treatments, while no differences in whelk counts occurred at FC and BB (Figure 11A). Sea stars were generally held at bay at all sites (i.e., densities were generally lower in invertebrate predator removals) but were more abundant at FC and BB (Figure 11B). These data reflect that most EM sea stars were the small six‐armed *Leptasterias* sp., which are more abundant in the mid zone and at FC and BB than at YB and SH (Figure 12). Small *P. ochraceus* were occasionally detected in EM experiments (Figure 12). When +P and −P densities did not differ, densities nonetheless were likely effectively reduced due to removal of invertebrate predators in −P plots during each monitoring period. Thus, invertebrate predator effect estimates were conservative, and likely even stronger than shown by increases in prey abundance in removal treatments.

#### 3.2.2 Exposed Low

**FIGURE 13:**
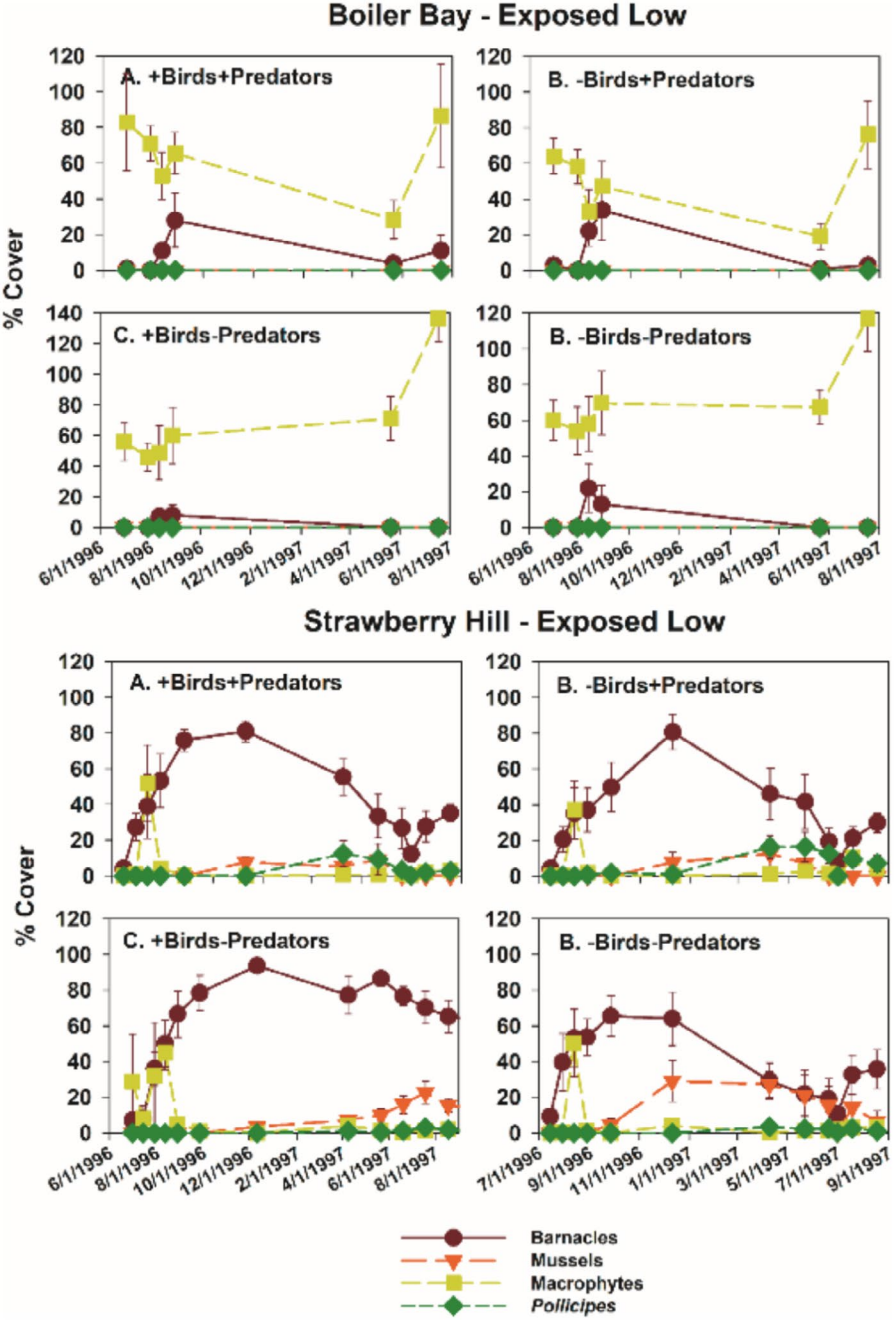
Time series of changes in the four major taxa responding to experimental manipulations in each treatment in Exposed Low zones at Boiler Bay and Strawberry Hill.

**FIGURE 14:**
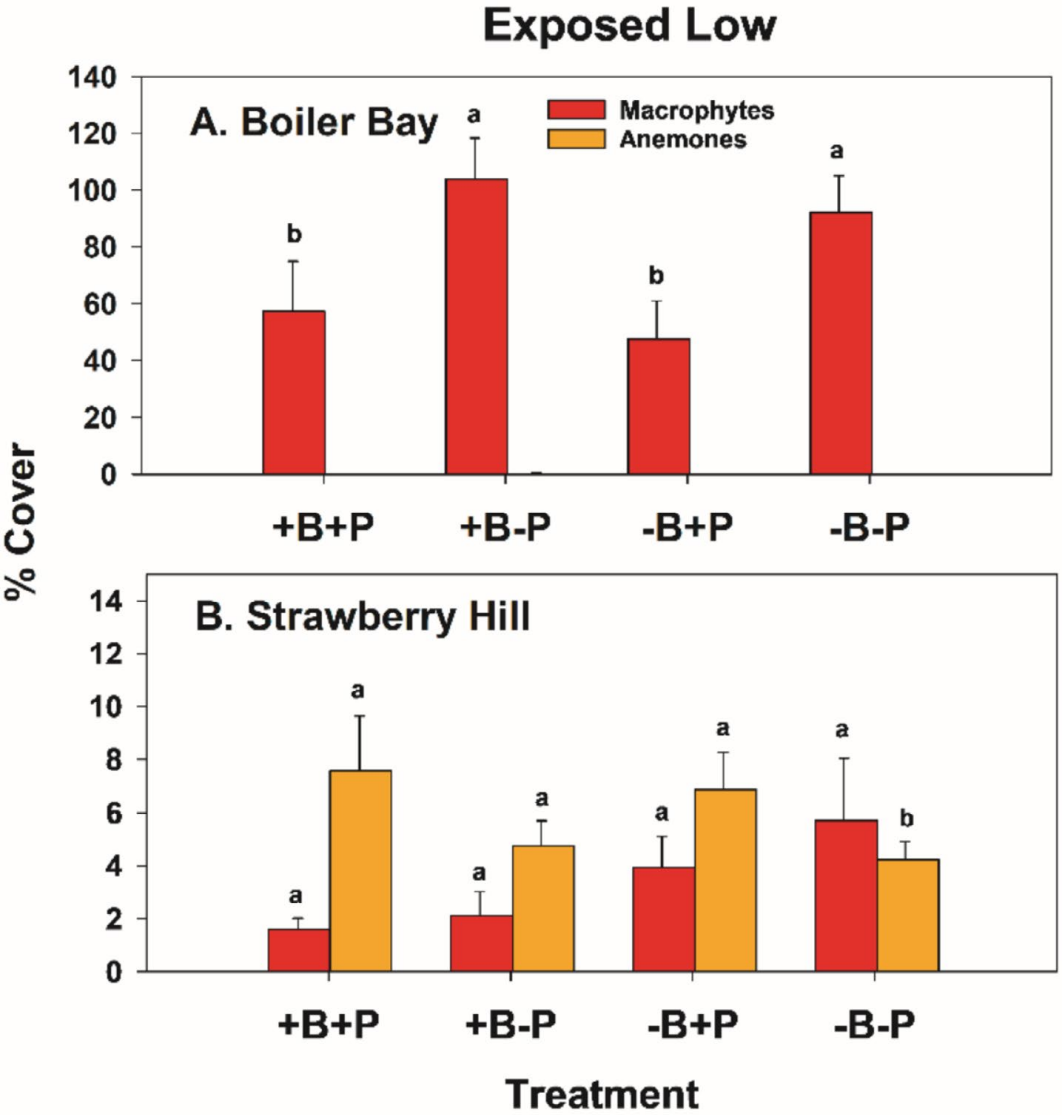
Mean and 1 SE (standard error) of percent cover of macrophytes and anemones in the four treatments at Boiler Bay (A) and Strawberry Hill (B). Cover of anemones at Boiler Bay was minuscule. Pairwise comparisons within (but not between) taxa were done using least squares contrasts. The lower‐case letters indicate within taxon differences among treatments, with no difference at *p* < 0.05 for those bars sharing the same letter.

As shown previously (Menge et al. 1994, 2004, 2015; Hacker et al. 2019; Gravem et al. 2024), community structure in EL zones differed rather dramatically between northern and southern sites. This difference was reflected in the results from the EL experiments at BB and SH (Figure 13). In all treatments, macrophytes dominated at BB and sessile invertebrates dominated at SH, both through time and in the second summer (1997) (Figures 13 and 14).

At the community level, site, sample time, and site × sample time explained the most variance (Table 6). While bird and invertebrate predator effects explained much less variance than these factors, invertebrate effects were always greater than bird effects in effect pairs (main effects, 5.18 vs. 1.48; interactions with site, 6.33 vs. 2.35; interactions with sample time, 3.83 vs. −3.90, respectively) (Table 6). Pairwise comparisons and nMDS ordinations show that invertebrate predation had effects on prey communities while bird predation did not (Table 7, Figure 10B). As in the EM experiments, dispersion in the EL experiments differed at the site × treatment scale (*p* = 0.001); this pattern was driven by between‐site variability (*p* = 0.001) since no differences occurred at the treatment scale (*p* = 0.57) (Table 5).

**TABLE 6:** Exposed Low intertidal zone: PERMANOVA test of effects of site, birds, and invertebrate predators on community structure in field experiments.

Source	df	SS	MS	Pseudo‐*F*	*p*(perm)	Unique permutations	Components of variation
Site	1	88,676	88,676	26.2	**0.003**	999	26.87
Bird effect	1	511.41	511.41	2.06	0.171	998	1.48
Pred effect	1	3760.6	3760.65	6.88	**0.009**	998	5.18
Sample time	11	93,463	8496.6	21.24	**0.001**	997	16.77
Site × bird effect	1	473.55	473.55	3.22	0.071	999	2.35
Site × predator effect	1	2687.2	2687.2	8.40	**0.025**	999	6.33
Site × sample time	5	16,936	3387.3	8.47	**0.001**	999	12.31
Bird × pred effect	1	141.18	141.18	0.65	0.551	999	−1.13
Bird × sample time	11	1992.6	181.15	0.45	0.977	998	−3.90
Pred × sample time	11	6725	611.36	1.53	0.059	998	3.83
Site × bird × pred effect	1	130.39	130.39	2.84	0.169	998	1.69
Site × bird × sample time	5	733.39	146.68	0.37	0.956	998	−5.07
Site × pred × sample time	5	1599.8	319.96	0.80	0.626	998	−2.86
Bird × pred × sample time	11	1504.1	136.74	0.34	0.994	999	−6.05
Site × bird × pred × sample time	5	227.78	45.557	0.11	0.992	998	−8.48
Residual	285	1.14E+05	400				20.00
Total	356	4.0034E+05					

*Note:* Sites were Boiler Bay and Strawberry Hill. The analysis was limited to the final months of the experiment, i.e., from April to September 1997. Community structure was the square‐root transformed abundance (percent cover) of acorn barnacles, gooseneck barnacles, mussels, anemones, macrophytes, and bare space. The analysis used a Bray–Curtis similarity resemblance matrix, with Type III (partial) sums of squares. Sample time was random, and site, bird, and predator effects were fixed, and the method was permutation of residuals under a reduced model. Boldface *p*‐values are significant at *p* < 0.05.

**TABLE 7:** Pair‐wise tests using PERMANOVA of effects of birds in invertebrate predator treatments and invertebrate predators in bird treatments in low exposed experiments at two sites.

Site	Bird effect		Predator effect	
	+P	−P	+B	−B
Boiler Bay	0.685	0.66	**0.014**	**0.027**
Strawberry Hill	0.314	0.313	**0.031**	**0.001**

*Note:* Data in the resemblance matrix were Bray–Curtis similarities based on square root transformed percent cover data. The analysis used Type III (partial) sums of squares with fixed effects summing to zero for mixed terms with 999 permutations of residuals under a reduced model. The analysis was based on 1997 data obtained in approximately monthly samples from April through September. The significance level is *p* < 0.05.

**FIGURE 15:**
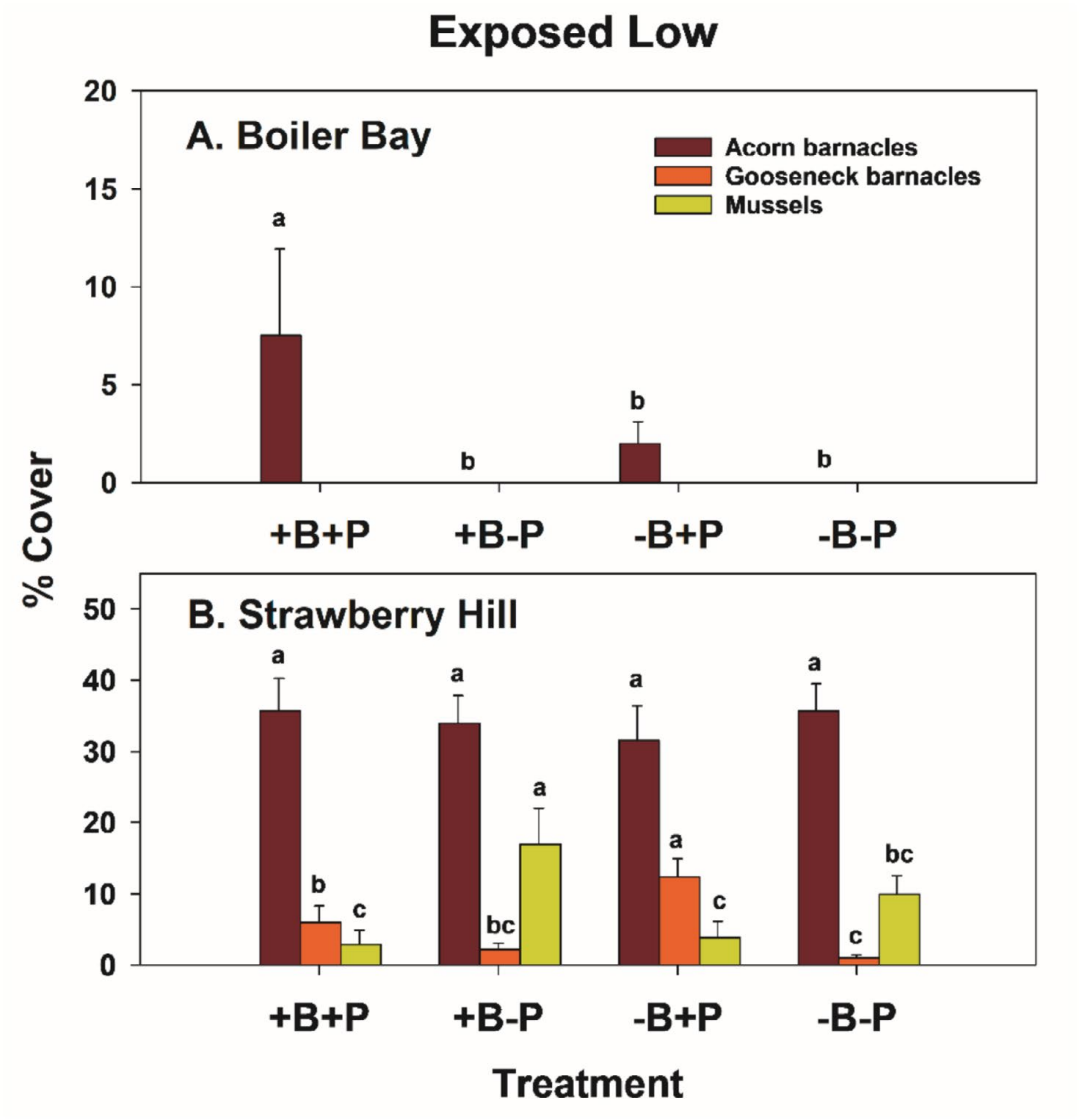
Mean and 1 SE (standard error) of percent cover of acorn barnacles, gooseneck barnacles, and mussels in the four low exposed treatments at Boiler Bay (A) and Strawberry Hill (B). Pairwise comparisons within (but not between) taxa were done using least squares contrasts. The lower‐case letters indicate within taxon differences among treatments, with no difference at *p* < 0.05 for those bars sharing the same letter.

In univariate tests in the EL zone, large between‐site differences in prey taxa dominated the analyses (Table S1), and a bird effect was found only for gooseneck barnacles at SH (Figure 15B, −B+P vs. +B treatments). At BB, invertebrate prey were sparse (< 8% cover of acorn barnacles, virtually no cover of gooseneck barnacles or mussels) (Figure 15). Mussel differences at SH suggested a moderately strong effect of invertebrate predators, with higher abundances in both −P treatments than in the +P treatments (Figure 15). Variance explained in EL experiments was 6.7, 8.2, and 22 times greater in −P than in −B treatments for acorn barnacles, gooseneck barnacles, and mussels, respectively (Table S1B).

**FIGURE 16:**
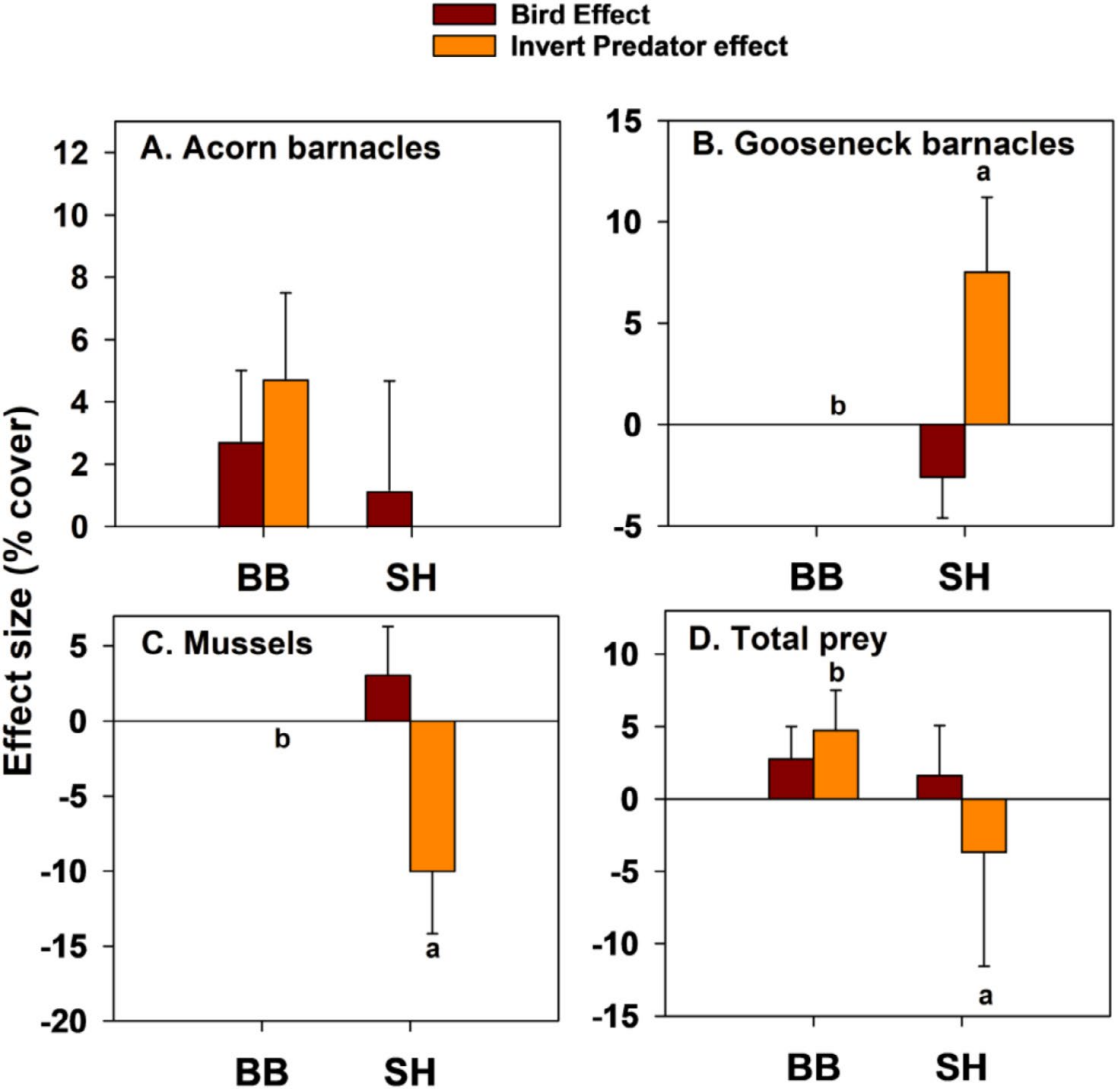
Effect size for exposed low experiments, quantified as the per replicate difference between +B vs. −B (purple bars) and +P vs. −P treatments (orange bars), for acorn (A) and gooseneck barnacles (B), mussels (C), and the sum of these three taxa (D). Bars sharing the same lower‐case letter were not different at *p* < 0.05. Pairwise comparisons employed linear contrasts. In all cases, bird effects did not differ between sites so no bars were coded with lower‐case letters.

Exposed low effect sizes were mostly positive for birds, while invertebrate predator effect sizes varied among taxa (Figure 16). EL effect sizes were also generally small, with most having absolute values of 5% or less, except for invertebrate predator effects on mussels at SH (Figure 16C).

**FIGURE 17:**
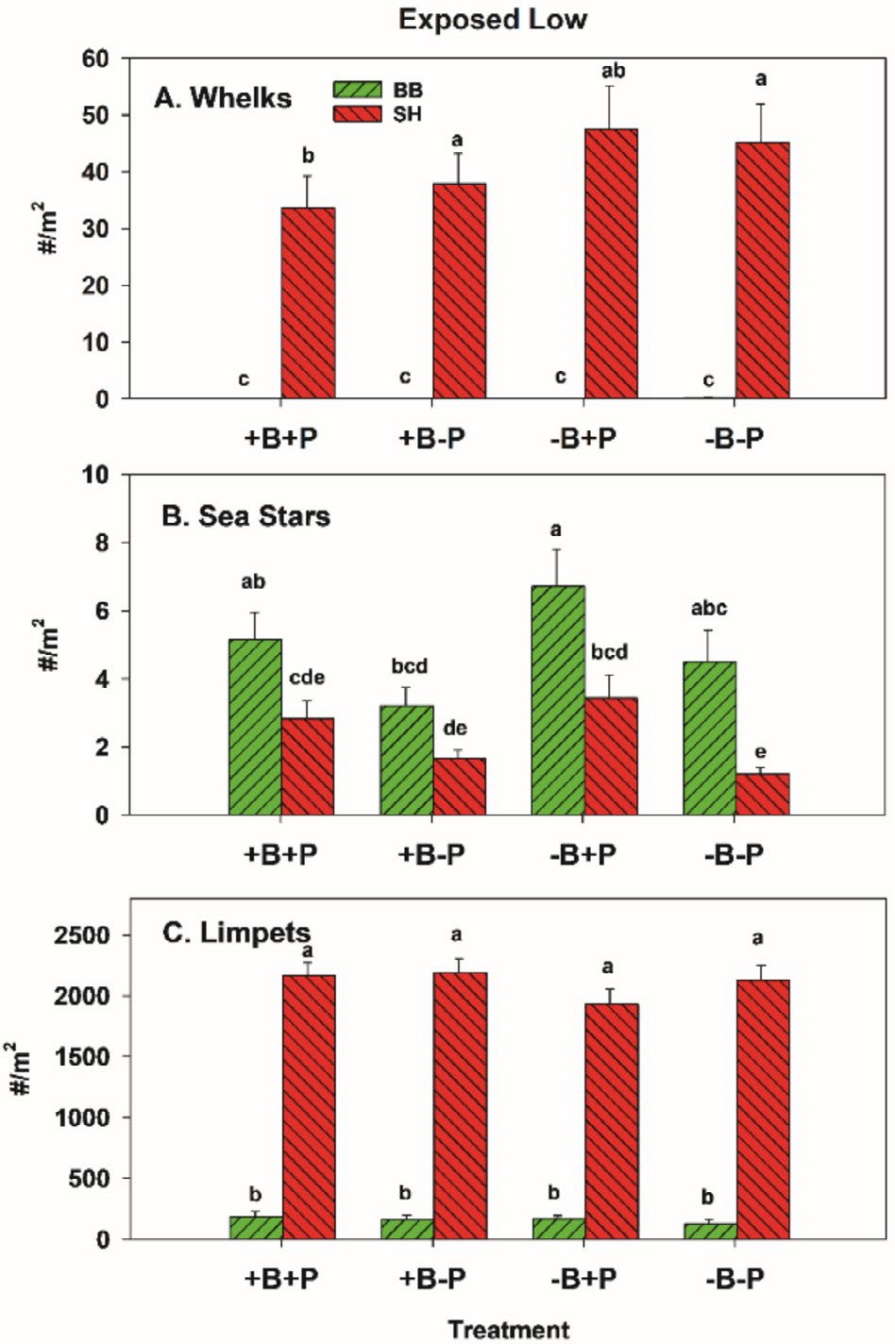
Overall mean number of whelks (A) and sea stars (B) either counted in (+P plots) or removed from (−P) plots by site. We also tracked limpet abundance (C) for comparison to possible indirect effects of manipulations on macrophytes. The lower‐case letters indicate differences among treatments and sites, with no difference at *p* < 0.05 for those bars sharing the same letter.

Whelks were abundant in the EL zone at SH and were almost absent at BB (Figure 17), where sea stars were the primary consumers. The trends in sea star abundance suggest removals from −P plots succeeded in reducing their average densities (Figures 12 and 17), but that reinvasions of −P plots by whelks were high at SH (Figure 17). No effects of birds or invertebrate predators were observed on limpet densities, but limpets were far denser at SH (Figure 17C).

#### 3.2.3 Protected Mid Experiments

**FIGURE 18:**
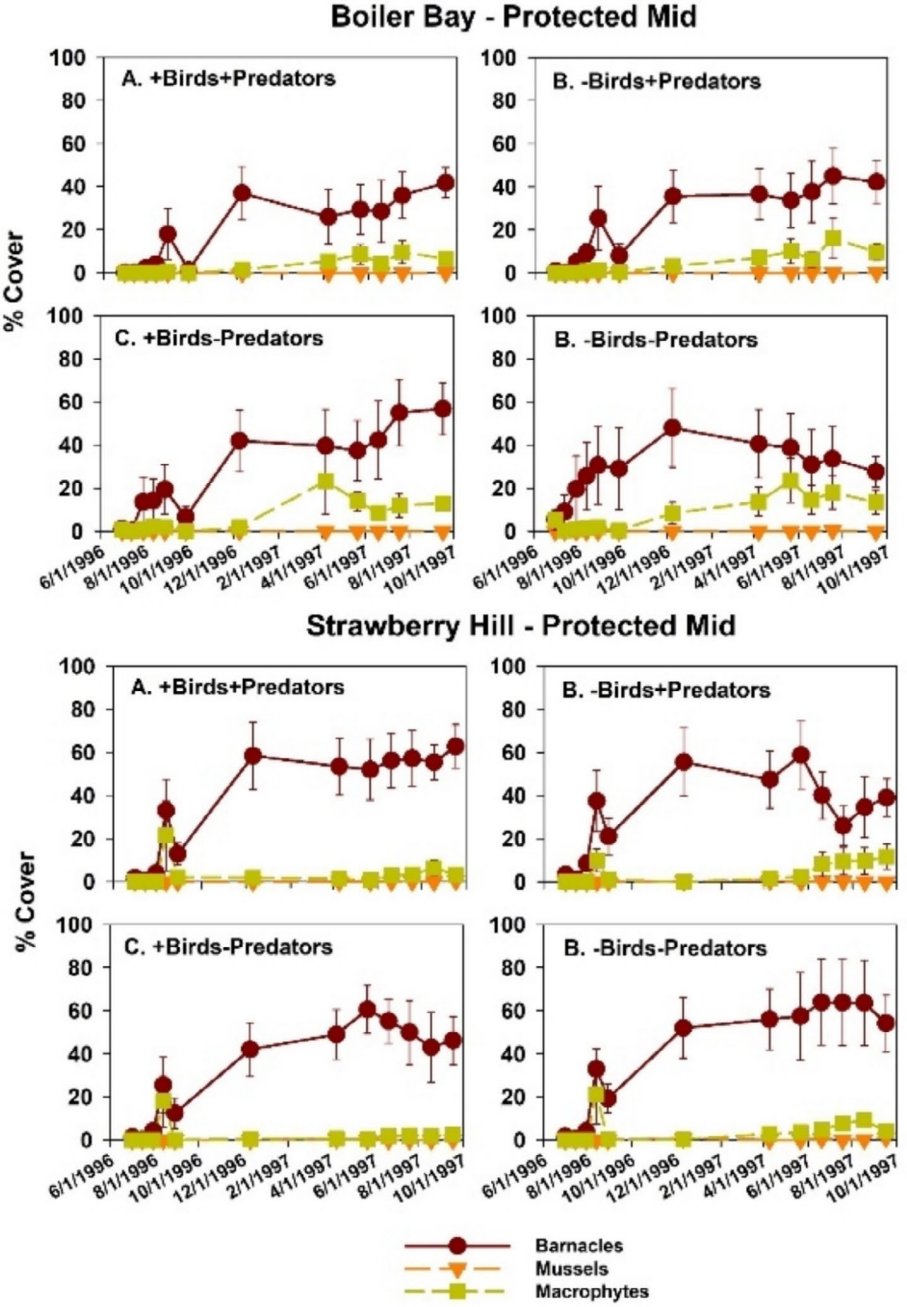
Time series of changes in the four major taxa responding to experimental manipulations in each treatment in Boiler Bay Protected Mid and Strawberry Hill Protected Mid zones.

Acorn barnacles and macrophytes were the only colonists in PM zone experiments (Figure 18). At the community level, results were like those in EM and EL zones: invertebrate predator effects were greater than bird effects, as indicated by components of variation (Table 8) and nMDS ordination (Figure 10C). Pairwise tests were mixed, with two of four significant effects of invertebrate predators in bird treatments and one of four bird effects in invertebrate predator treatments (Table 9). In this case, dispersions were different at all group levels, site × treatment (*p* = 0.001), site (*p* = 0.015), and treatment (*p* = 0.001) (Table 5).

**TABLE 8:** Protected Mid intertidal zone: PERMANOVA test of effects of site, birds, and invertebrate predators on community structure in field experiments.

Source	df	SS	MS	Pseudo‐*F*	*p*(perm)	Unique permutations	Components of variation
Site	1	14,944	14,944	12.46	**0.005**	999	7.58
Bird effect	1	617.6	617.6	5.70	**0.048**	999	1.46
Pred effect	1	1815.6	1815.6	63.17	**0.001**	999	2.73
Sample time	11	1.2305e+05	11,186	29.64	**0.001**	997	16.46
Site × bird effect	1	180.85	180.85	1.45	0.268	999	0.69
Site × predator effect	1	1057.4	1057.4	4.99	**0.033**	997	2.66
Site × Sample time	11	13,191	1199.2	3.18	**0.001**	998	6.42
Bird × pred effect	1	244.75	244.75	7.94	0.082	999	1.34
Bird × sample time	11	1192	108.37	0.29	0.99	999	−3.67
Pred × sample time	11	315.91	28.719	0.076	1	998	−4.18
Site × bird × pred effect	1	1421.2	1421.2	5.23	0.055	999	4.38
Site × bird × sample time	11	1370	124.54	0.33	0.986	999	−5.04
Site × pred × sample time	11	2333	212.09	0.56	0.895	999	−4.07
Bird × pred × sample time	11	338.65	30.786	0.08	1	998	−5.90
Site × bird × pred × sample time	11	2989	271.73	0.72	0.752	998	−4.60
Residual	383	1.4456e+05	377.44				19.43
Total	478	3.0947e+05					

*Note:* Sites were Boiler Bay and Strawberry Hill. The analysis was limited to the final months of the experiment, i.e., from April to September 1997. Community structure was the square‐root transformed abundance (percent cover) of acorn barnacles, gooseneck barnacles, mussels, anemones, macrophytes, and bare space. The analysis used a Bray–Curtis similarity resemblance matrix, with Type III (partial) sums of squares. Sample time was random, and site, bird, and predator effects were fixed, and the method was permutation of residuals under a reduced model. Boldface *p*‐values indicate significance at *p* < 0.05.

**TABLE 9:** Pair‐wise tests using PERMANOVA of effects of birds in invertebrate predator treatments and invertebrate predators in bird treatments in mid protected experiments at two sites.

Site	Bird effect		Predator effect	
	+P	−P	+B	−B
Boiler Bay	0.468	0.103	**0.036**	0.343
Strawberry Hill	**0.026**	0.12	0.457	**0.013**

*Note:* Data in the resemblance matrix were Bray–Curtis similarities based on square root transformed percent cover data. The analysis used Type III (partial) sums of squares with fixed effects summing to zero for mixed terms with 999 permutations of residuals under a reduced model. The analysis was based on 1997 data obtained in approximately monthly samples from April through September. The significance level is *p* < 0.05.

**FIGURE 19:**
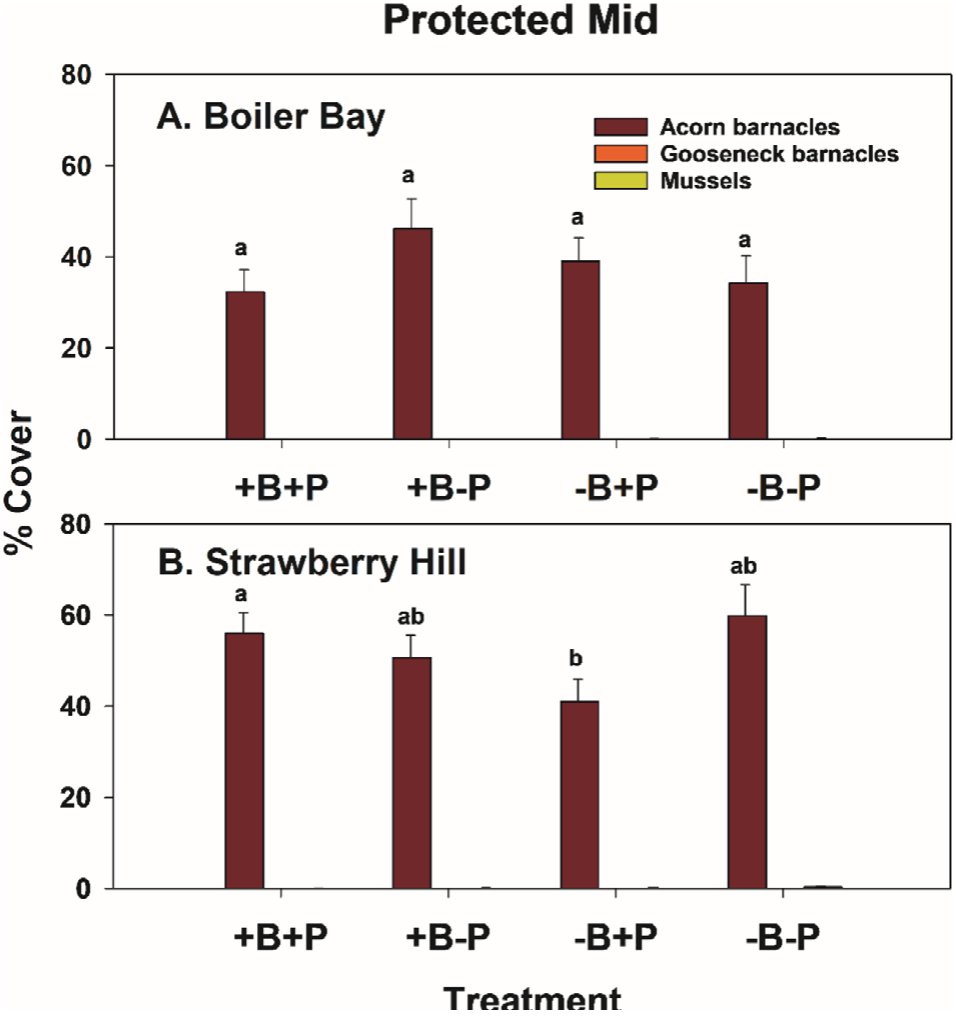
Mean and 1 SE (standard error) of percent cover of protected mid zone acorn barnacles, gooseneck barnacles, and mussels in the four treatments at Boiler Bay (A) and Strawberry Hill (B). Pairwise comparisons within (but not between) taxa were done using least squares contrasts. Gooseneck barnacle and mussel abundance were very low and are barely evident in the figure. The lower‐case letters indicate within taxon differences among treatments, with no difference at *p* < 0.05 for those bars sharing the same letter.

In univariate tests in the PM zone, all factors apparently had effects (site × bird × predator interaction *p* = 0.013) (Table S1C), but this was likely mostly a site effect since neither predator type had effects on acorn barnacles at BB, and acorn barnacles were more abundant at SH (Figure 19). At SH in +P treatments, a bird effect was detected, but again, contrary to expectation, barnacles were more, not less, abundant in bird presence and did not differ in +B−P vs. −B−P plots (Figure 19B). Consistent with these results, effect sizes of birds and invertebrate predators were essentially 0 at all sites (data not shown).

**FIGURE 20:**
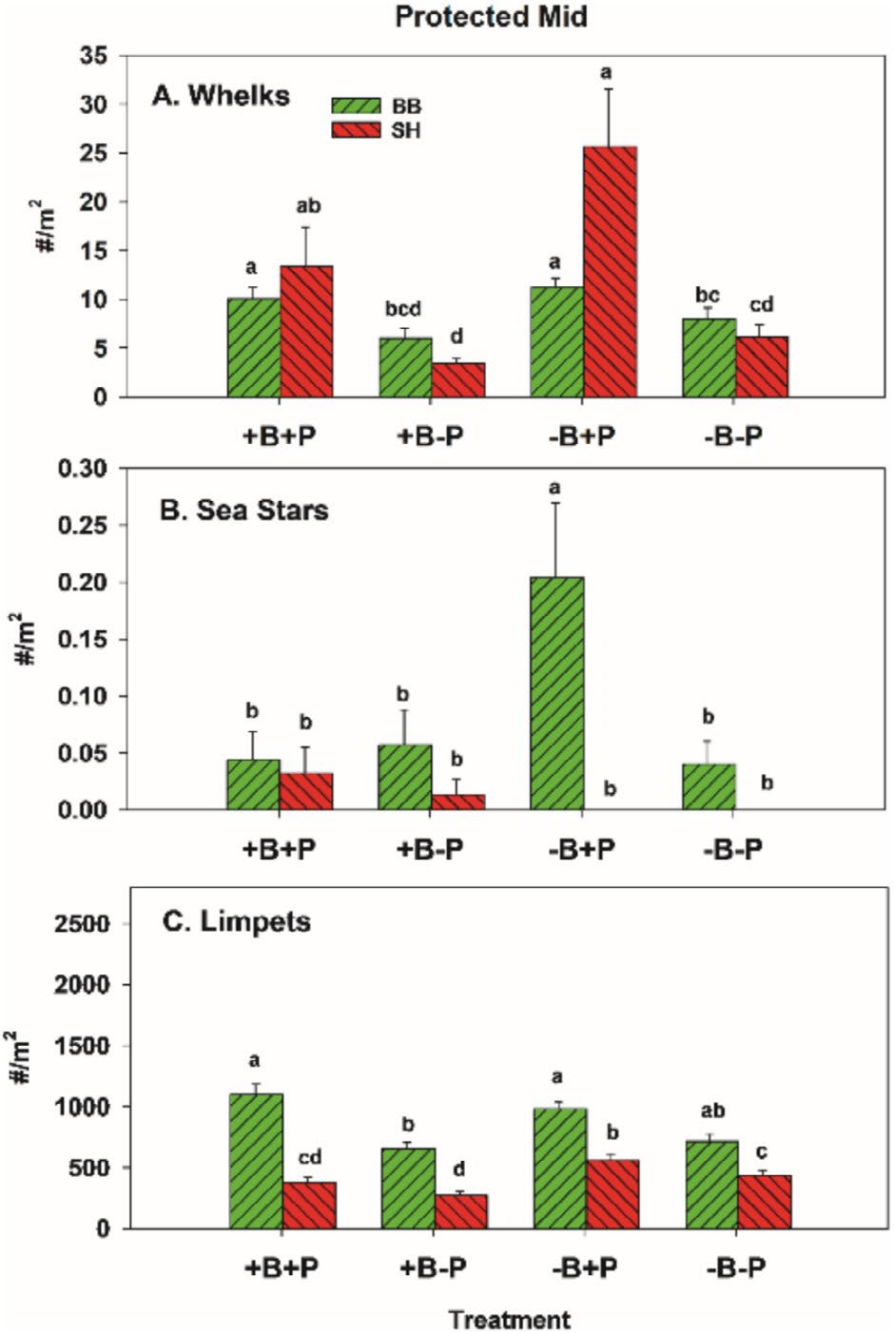
Overall mean number of whelks (A) and sea stars (B) either counted in (+P plots) or removed from (−P) plots by site in protected mid experiments. We also tracked limpet abundance (C) for comparison to possible indirect effects of manipulations on macrophytes. The lower‐case letters indicate differences among treatments and sites, with no difference at *p* < 0.05 for those bars sharing the same letter.

Whelk removals were effective at both sites (Figure 20), but sea stars were sparse in PM areas (Figure 20B). So, despite a higher density at BB in the −B+P treatment, this difference is likely of minimal importance. Limpet densities varied inconsistently among treatments at both sites (Figure 20C).

### 3.3 Bird Effects on Invertebrate Predators

Several trends suggested that bird predation may have affected invertebrate predator abundance (e.g., Figures 12 and 17). That is, whelks tended to be more abundant in bird absence plots, at least at YB and SH. In EM experiments, the site, invertebrate predator removal, and site × invertebrate predator removal treatments were all significant, but bird treatments were not and explained little variance (Table S2). Similarly, in EL experiments, only site and invertebrate predator removal had effects on sea stars. Tests of effects on limpets revealed only site differences, with no effect of either type of predator on limpet densities (analysis not shown).

## 4 Discussion

Bird predation effects were minimal in these experiments and weaker than invertebrate predator effects. Through interactions with site, sample time, and invertebrate predators, weak bird effects on community structure were detected in the EM and PM zones. For invertebrate prey, bird effects were detected for all three prey types in the EM zone, for gooseneck barnacles in the EL zone, and through an interaction with site and invertebrate predators at PM sites. However, these effects were often opposite to what was expected, varied by site, generally explained little variation, and did not occur consistently in +B and −B comparisons. For example, at SH the gooseneck barnacle *Pollicipes polymerus* was more abundant in EL experiments in the −B+P than in the +B+P treatment but not in the −B−P vs. the +B−P treatment (Figure 15B). Apparent bird effects on acorn barnacles (Figure 15A, BB EL; Figure 19B, SH PM) were likely spurious or results of an indirect interaction with other space occupants since the barnacles were more, not less abundant in the presence of birds.

In contrast, and consistent with prior results, effects of invertebrate predators were stronger and occurred more consistently across sites and zones despite high rates of reinvasion after removal by some taxa at some sites. The effects of these consumers were strongest in the EM zone (e.g., Figure 9) and at Cape Foulweather (FC, BB) sites, likely an example of “diffuse” predation (e.g., Menge et al. 1994; Robles 1997; Menge 2003; Heupel et al. 2014) since multiple predator species (2 whelks and 2 sea stars) were abundant at these sites (Figures 11 and 12). The relatively low abundances of both sea star species in the EM zone at Cape Perpetua (YB, SH) sites (Figure 11) are consistent with historical data for the mid intertidal. Low abundances in the PM zones at BB and SH are also consistent with previous data for *Leptasterias* sp. and *P. ochraceus* but not with more recent data for *P. ochraceus* in the EL zone at SH (e.g., Menge et al. 2004, 2016). A more recent study to be reported elsewhere will test if this trend has persisted to the 2020's.

Compared with Wootton's (1992, 1994, 1997) results which showed large bird effects (e.g., after 1 year *P. polymerus* cover was ~60% in −B plots and 10% in +B plots), the weak to non‐existent effects detected here were surprising. Since the present study was conducted just a few years after Wootton's, why the difference? I hypothesize that the difference is based on whether bird populations are residents vs. transients. Tatoosh Island is just off Cape Flattery in Washington state, requires tribal and Coast Guard permission to reach, and, like many coastal offshore islands, has resident gull, oystercatcher, and crow populations (Paine et al. 1990; Parrish et al. 2001). Wootton's (1997) census data indicate that the gull colony size was huge compared to the two offshore sites in Oregon (2280 vs. 57 and 276). In contrast, all the Oregon study sites were readily accessible (to humans) mainland sites. Although most have one or two pairs of resident oystercatchers (Liebezeit et al. 2020), none of them have resident gull or crow populations (Author's personal observations). Birds, especially gulls, regularly visit these sites (Figure 4, Author's personal observations), are often observed consuming invertebrates, and, as noted in the Introduction, can leave evidence of what they had eaten. However, the numbers of gulls observed per visit in 2018–19 averaged < 5/100 m shoreline (Figure 4) for all sites but YB, where visitation rates were high (~18/100 m of shoreline). Crow and oystercatcher numbers were far less, ~1–2/100 m shoreline, and densities of these taxa on Tatoosh were comparable to those in Oregon (Figure 4). Note that despite gull counts being four times higher at YB than at SH, weak bird effects on prey were detected only at SH (Table 4).

These comparisons raise the question of why bird predation was so weak at YB, which actually had higher densities of sea gulls than did Tatoosh Island, where bird predation was strong. The major difference between Tatoosh and all the Oregon mainland sites was that Tatoosh had a large resident population, while birds at Oregon mainland sites were non‐residents. I thus hypothesize that the difference in bird predation between experiments reported here versus those on Tatoosh is that bird predation is much more intense at sites with resident bird populations versus at sites where birds are transient. Such differences are likely general since offshore rocks in Oregon also had relatively high abundances of gulls (Figure 3, Table 1). This hypothesis could be tested by comparing bird predation effects at mainland versus offshore island sites.

Another possibility is that human disturbance may account for differences between Tatoosh and the Oregon mainland sites. As noted above, humans (usually fishers or tidepoolers) were observed in 14 of 47 surveys. I tested this possibility for gulls and crows; however, I found no differences in bird density associated with human presence (three‐way ANOVA: humans as a main effect, *p* = 0.77, interacting with site, species or both, *p* = 0.46, 0.99, and 0.55 respectively, *n* = 76, adj. *R* ^2^ = 0.47, model *p* < 0.0001). Few instances of oystercatchers observed with humans present were made, but for FC and YB, no human effect was detected (two‐way ANOVA: humans as a main effect *p* = 0.23, interacting with site *p* = 0.11, *n* = 18, adj. *R* ^2^ = 0.17). These results suggest that human traffic at my sites was insufficient to alter bird presence.

Other differences between the two experiment sets that could influence results include experiment length and initial experimental conditions. Wootton's (1994) experiment ran for 24 months vs. ~17 months for my Oregon experiments. However, gooseneck barnacle abundance was nearly identical after 1 vs. 2 years in Wootton's study, so experiment length per se does not seem a likely explanation. Perhaps more important was that his experiments started in 1.5‐year‐old gaps in mussel beds on space already occupied by recently settled *P. polymerus* while the Oregon experiments started on newly cleared space; i.e., his experiments were started at a later successional stage. In Oregon (Figures 5, 6, 13 and 18), first‐year results were early successional, featuring high abundances of acorn barnacles (mostly *B. glandula* ) and macrophytes. *B. glandula* abundance usually declined over the 1996–1997 winter and early spring, when longer‐lived prey species began increasing. The original intent was to run experiments longer, but fall and winter storm removal of cages effectively terminated the experiment.

In my experiments, the stronger effects of invertebrate predation compared to birds were expected since historically whelks and sea stars have often been documented to be strong interactors in rocky intertidal communities (Connell 1961; Dayton 1971; Paine 1971, 1974; Menge 1976; Menge et al. 1994, 1999, 2004; Navarrete and Menge 1996; Navarrete 1996; Berlow 1997; Wootton 2002; Novak 2010). However, the stronger effect of invertebrate predation in the EM vs. the EL intertidal (e.g., Figures 9 and 15) was unexpected. Prior studies, including Paine's classic experiments (1966, 1974) had shown strong sea star (i.e., *P. ochraceus* ) predation effects in the low zone, which is where the bulk of *P. ochraceus* populations live and feed. Thus, the weak effect of predation in EL sites (Figure 15) was puzzling.

Two factors may explain this result: (1) the early successional aspect of the first 9 months of the experiment and (2) spatio‐temporal differences in the abundance of small predators. Acorn barnacles were the dominant prey during the first 9 months of the experiment, so they were likely more attractive to small predators than to *P. ochraceus* , which favors larger mussels (Paine 1974, 1976). In addition, primary differences in predator composition between zones were that (1) whelks were far more abundant in the EL at SH than at BB, with similar but smaller differences in the EM, while (2) sea stars were more abundant in the EM, at least at BB, than in the EL (Figures 11 and 17). This difference was due largely to higher *Leptasterias* sp. abundances at northern sites (Figure 12). Thus, like Navarrete (1996), Berlow (1997) and Wootton (2002), small consumer predation was evidently stronger early in the experiment.

Predation effect differences also were observed along the wave‐exposed (stronger predation) to wave‐protected (weaker predation) gradient in the mid zone at BB and SH. Specifically, (1) all invertebrate predator taxa were less abundant in wave‐protected areas, especially sea stars (Figures 12 and 20); (2) wave‐protected areas are much less productive in prey recruitment and growth (Menge et al. 1994); and (3) protected areas also have much lower abundances of gooseneck barnacles (op. cit.) and are likely unattractive places for birds to forage.

### 4.1 Generality

Impacts of bird predation on rocky intertidal prey populations have been investigated on shores around the world including Canada (Hamilton 2000; Hamilton and Nudds 2003; Cheverie et al. 2014), California (Meese 1993), Japan (Hori and Noda 2001), New England (Ellis et al. 2007), Oregon (Marsh 1986a, 1986b), South Africa (Hockey and Bosman 1988), the UK (Feare 1971), and Washington (Wootton 1992, 1994). Many showed strong effects on prey populations, variously including limpets, mudsnails, amphipods, mussels, sea urchins, gooseneck barnacles, and whelks. Besides Wootton's (1992, 1994, 1997) studies, particularly relevant examples were Hamilton's (2000) demonstration of strong reduction of mussel abundance by eiders, Meese's (1993) study showing strong negative effects of gulls on gooseneck barnacles, and Ellis et al.'s (2007) documentation of a gull‐driven trophic cascade through predation on crabs. Thus, my study showing weak effects across multiple sites, different wave exposures, and different zones appears exceptional. However, in South Africa, Hockey and Bosman (1988) found spatial differences in the effects of African Black Oystercatcher ( *Haematopus moquini* ) on limpets (*Patella* spp.), with large effects on islands with dense oystercatcher populations and small effects on the mainland with sparser oystercatcher populations. These differences are consistent with the differences between Oregon (mainland) and Tatoosh Island (island) results.

Despite decades of research on terrestrial avian ecology, attempts to experimentally test their effects on prey and associated communities were slow to draw the attention of ecologists. Early examples include Atlegrim (1989) who found direct effects of bird predation on insect abundance, with indirect positive effects on bilberry plants; Bock et al. (1992) who found that bird predation reduced grasshopper abundance, though in this case, no indirect effect on plants was detected; and Marquis and Whelan (1994) whose exclusion experiments showed that insectivorous bird predation on herbivorous insects indirectly enabled increased growth in white oak. In general, experimental exclusion of terrestrial birds leads to increases in arthropods, both predatory and herbivorous, and in some cases, effects cascade down, leading to increased vegetation biomass or growth (e.g., Strong et al. 2000; Murakami and Nakano 2002; Gruner 2004; Schwenk et al. 2010; see metanalyses in Van Bael et al. 2008 and Mooney et al. 2010). Thus, birds can exert top‐down effects on their prey, sometimes strongly enough to induce trophic cascades. An important goal will be to determine the conditions that underlie such variation.

## 5 Conclusion

In experiments of comparable design to those of Wootton (1994) on Tatoosh Island, Washington state, I tested shorebird predation crossed with invertebrate predation effects on prey (barnacles, mussels) and associated species. I found weak effects of birds in wave‐exposed mid‐intertidal habitats but no effects in low intertidal or more protected habitats. Invertebrate predators, primarily whelks and sea stars, had much stronger effects that were consistent across sites in the mid intertidal but surprisingly relatively weak in low intertidal areas. Hypothetically, the difference between Wootton's (1994) study showing strong bird effects, especially on gooseneck barnacles, and this study showing weak to no bird effects may reflect differences between sites with resident, abundant bird populations (Tatoosh Island) and sites with transient, sparser bird activity. Whether or not this explains differences in bird effects, a focus of future research in both marine and terrestrial environments should be to identify the factors that underlie variation in the strength of this important group of consumers.

## Author Contributions

**Bruce A. Menge:** conceptualization (equal), data curation (equal), formal analysis (equal), funding acquisition (equal), investigation (equal), methodology (equal), project administration (equal), resources (equal), supervision (equal), validation (equal), visualization (equal), writing – original draft (equal), writing – review and editing (equal).

## Disclosure

Statement on inclusion: Field work and discussion of the project included assistance and input from multiple female members of my laboratory, including Dr. Halpin.

## Conflicts of Interest

The author declares no conflicts of interest.

## Supporting information


Appendix S1

## Data Availability

Data from this study are available at https://doi.org/10.6084/m9.figshare.28464641.v1.
