# Supplementary material for: Impacts of Birds vs. Invertebrate Predators on Rocky Intertidal Community Structure
Source: Ecol Evol. 2025 Mar 18;15(3):e71121. doi: 10.1002/ece3.71121 (PMC11919729; doi:10.1002/ece3.71121)
Supplement: Supplementary file 1 — Appendix S1 [file ECE3-15-e71121-s001.docx]

**APPENDIX S1**

**Title: Impacts of Birds vs. Invertebrate Predators on Rocky Intertidal Community Structure**


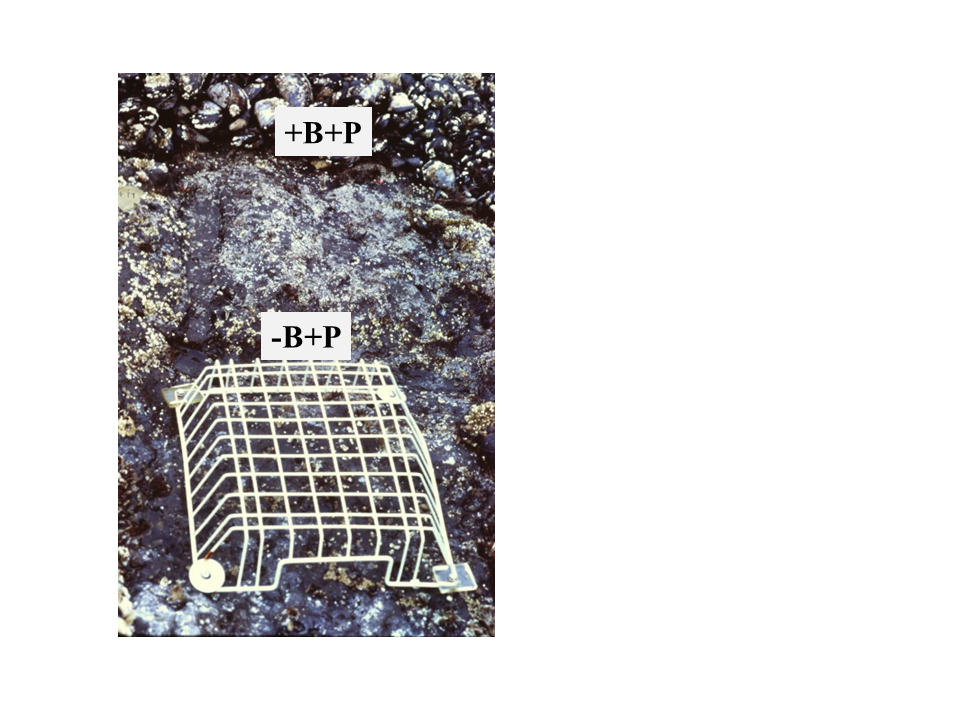


Figure S1. Photograph showing complete exclusion cage and adjacent control plot. Treatment codes are +B and -B = birds present and absent, and +P = invertebrate predators present. Photo by B. Menge.


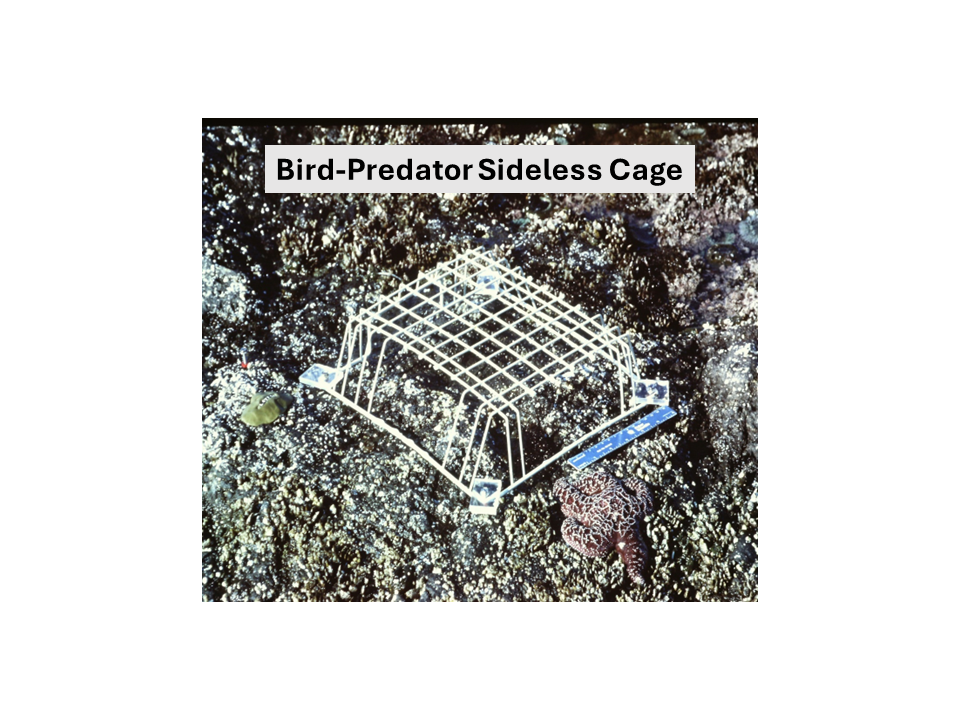


Figure S2. Example of exclusion cage with sides removed to facilitation entry by large sea stars (e.g., the purple *Pisaster ochraceus* next to the ruler). Photo by B. Menge.


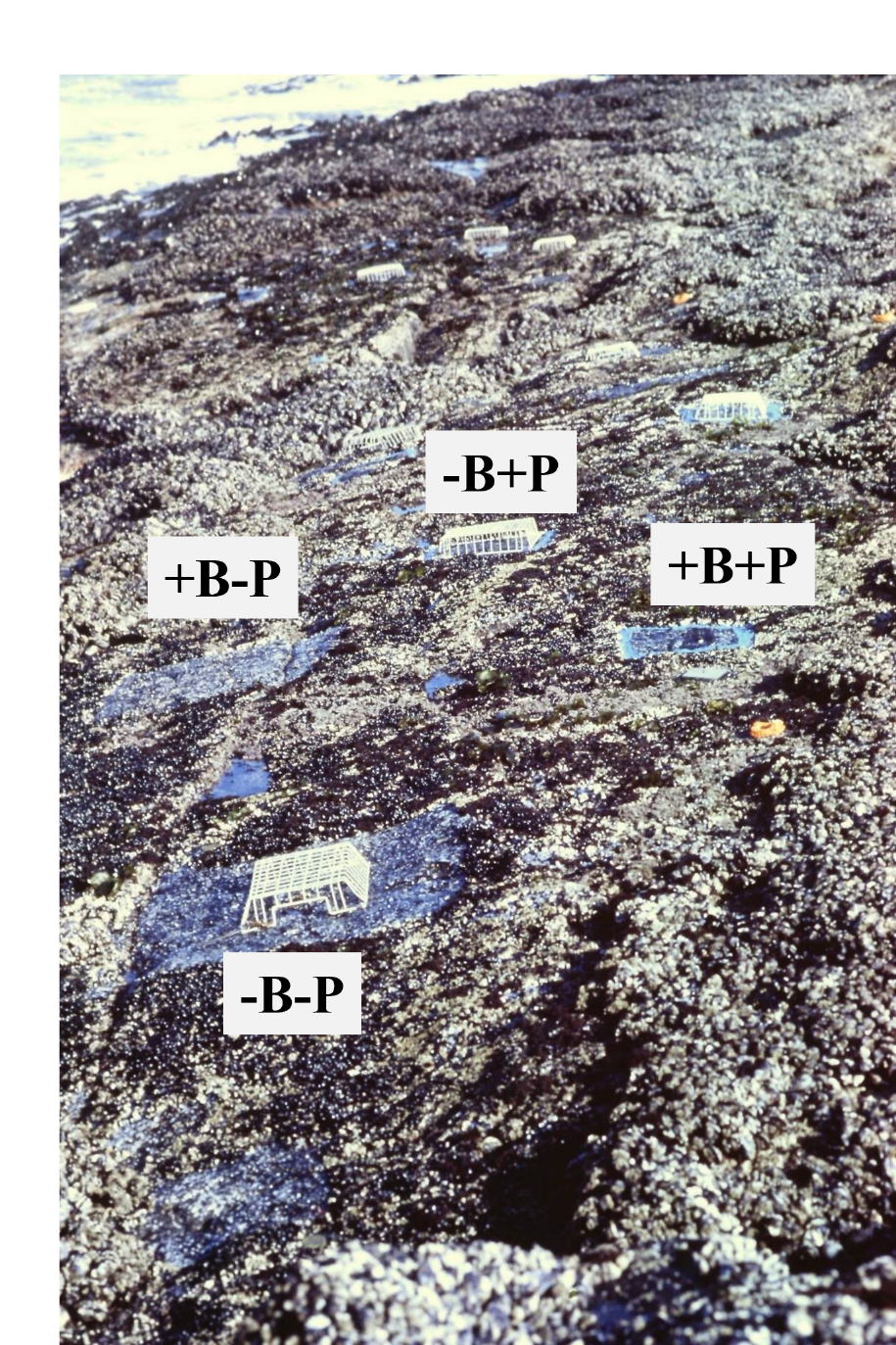


Figure S3. Partial array of treatments at Boiler Bay, showing the prey-free zone around the -B-P cage and the similar-sized cleared plot for the +B-P plot above it. No prey-free zone was cleared around the -B+P and +B+P plots. Photo by B. Menge.

Table S1. Univariate tests of effects of site, bird predation, and invertebrate predation (all fixed) on abundance of acorn barnacles, gooseneck barnacles, and mussel in experiments at exposed mid, exposed low, and protected mid sites. No results are shown for gooseneck barnacles at protected mid sites because of lack of colonization by this taxon. No results are shown for protected mid mussels because no factor had a significant effect on mussel abundance. Boldface p values indicate factors significant at p < 0.05 or less. Variance explained is eta-squared (SS_effect_/SS_total_) for each source and adjusted R^2^ for the full model.

| **Exposure, Zone** | **Taxon** | **Source** | **df** | **SS** | **F** | **p** | **Variance explained (proportion)** |
| --- | --- | --- | --- | --- | --- | --- | --- |
| 1. **Exposed Mid** | **Acorn barnacles** | Site | 3 | 112.434 | 41.38 | **<0.0001** | 0.225 |
|  |  | Birds | 1 | 9.537 | 10.53 | **0.0013** | 0.019 |
|  |  | Site x Birds | 3 | 4.992 | 1.84 | 0.14 | 0.010 |
|  |  | Predators | 1 | 19.195 | 21.19 | **<0.0001** | 0.038 |
|  |  | Site x Predators | 3 | 10.656 | 3.92 | **0.009** | 0.021 |
|  |  | Birds x Predators | 1 | 0.7001 | 0.77 | 0.38 | 0.001 |
|  |  | Site x Birds x Predators | 3 | 1.420 | 0.52 | 0.67 | 0.003 |
|  |  | Model | 15 | 160.960 | 11.85 | **<0.0001** |  |
|  |  | Error | 373 | 337.828 |  |  |  |
|  |  | C. Total | 388 | 498.788 |  |  | 0.295 |
|  |  |  |  |  |  |  |  |
|  | **Gooseneck barnacles** | Site | 3 | 70.662 | 26.44 | **<0.0001** | 0.155 |
|  |  | Birds | 1 | 6.595 | 7.40 | **0.007** | 0.014 |
|  |  | Site x Birds | 3 | 2.927 | 1.10 | 0.35 | 0.006 |
|  |  | Predators | 1 | 5.856 | 6.57 | **0.011** | 0.013 |
|  |  | Site x Predators | 3 | 30.510 | 11.42 | **<0.0001** | 0.067 |
|  |  | Birds x Predators | 1 | 0.101 | 0.11 | 0.74 | 0.0002 |
|  |  | Site x Birds x Predators | 3 | 6.764 | 2.53 | **0.06** | 0.015 |
|  |  | Model | 15 | 122.977 | 9.20 | **<0.0001** |  |
|  |  | Error | 373 | 332.269 |  |  |  |
|  |  | C. Total | 388 | 455.245 |  |  | 0.241 |
|  |  |  |  |  |  |  |  |
|  | **Mussels** | Site | 3 | 180.827 | 65.92 | **<0.0001** | 0.264 |
|  |  | Birds | 1 | 0.292 | 0.32 | 0.57 | 0.0004 |
|  |  | Site x Birds | 3 | 9.032 | 3.29 | **0.021** | 0.013 |
|  |  | Predators | 1 | 105.210 | 115.07 | **<0.0001** | 0.154 |
|  |  | Site x Predators | 3 | 40.790 | 14.87 | **<0.0001** | 0.060 |
|  |  | Birds x Predators | 1 | 4.255 | 4.65 | **0.032** | 0.006 |
|  |  | Site x Birds x Predators | 3 | 4.950 | 1.80 | 0.15 | 0.007 |
|  |  | Model | 15 | 342.899 | 25.00 | **<0.0001** |  |
|  |  | Error | 373 | 341.040 |  |  |  |
|  |  | C. Total | 388 | 683.939 |  |  | 0.481 |
|  |  |  |  |  |  |  |  |
| 1. **Exposed Low** | **Acorn barnacles** | Site | 1 | 237.277 | 262.70 | **<0.0001** | 0.592 |
|  |  | Birds | 1 | 1.530 | 1.69 | 0.19 | 0.004 |
|  |  | Site x Birds | 1 | 0.019 | 0.02 | 0.88 | 0.00005 |
|  |  | Predators | 1 | 4.964 | 5.50 | **0.02** | 0.012 |
|  |  | Site x Predators | 1 | 5.822 | 6.45 | **0.012** | 0.015 |
|  |  | Birds x Predators | 1 | 0.387 | 0.43 | 0.51 | 0.001 |
|  |  | Site x Birds x Predators | 1 | 0.569 | 0.63 | 0.43 | 0.001 |
|  |  | Model | 7 | 250.873 | 39.68 | **<0.0001** |  |
|  |  | Error | 166 | 149.932 |  |  |  |
|  |  | C. Total | 173 | 400.805 |  |  | 0.610 |
|  |  |  |  |  |  |  |  |
|  | **Gooseneck barnacles** | Site | 1 | 29.280 | 39.68 | **<0.0001** | 0.148 |
|  |  | Birds | 1 | 1.070 | 1.45 | 0.23 | 0.005 |
|  |  | Site x Birds | 1 | 1.070 | 1.45 | 0.23 | 0.005 |
|  |  | Predators | 1 | 8.108 | 10.99 | **0.001** | 0.041 |
|  |  | Site x Predators | 1 | 8.108 | 10.99 | **0.001** | 0.041 |
|  |  | Birds x Predators | 1 | 3.592 | 4.87 | **0.029** | 0.018 |
|  |  | Site x Birds x Predators | 1 | 3.592 | 4.87 | **0.029** | 0.018 |
|  |  | Model | 7 | 75.224 | 14.56 | **<0.0001** |  |
|  |  | Error | 166 | 122.481 |  |  |  |
|  |  | C. Total | 173 | 197.705 |  |  | 0.354 |
|  |  |  |  |  |  |  |  |
|  | **Mussels** | Site | 1 | 24.898 | 16.38 | **<0.0001** | 0.080 |
|  |  | Birds | 1 | 0.425 | 0.28 | 0.60 | 0.001 |
|  |  | Site x Birds | 1 | 0.425 | 0.28 | 0.60 | 0.001 |
|  |  | Predators | 1 | 6.937 | 4.56 | **0.034** | 0.022 |
|  |  | Site x Predators | 1 | 6.937 | 4.56 | **0.034** | 0.022 |
|  |  | Birds x Predators | 1 | 1.143 | 0.75 | 0.39 | 0.004 |
|  |  | Site x Birds x Predators | 1 | 1.143 | 0.75 | 0.39 | 0.004 |
|  |  | Model | 7 | 57.437 | 5.40 | **<0.0001** |  |
|  |  | Error | 166 | 252.382 |  |  |  |
|  |  | C. Total | 173 | 309.819 |  |  | 0.151 |
|  |  |  |  |  |  |  |  |
| 1. **Protected Mid** | **Acorn barnacles** | Site | 1 | 11.240 | 15.83 | **<0.0001** | 0.067 |
|  |  | Birds | 1 | 1.377 | 1.94 | 0.17 | 0.008 |
|  |  | Site x Birds | 1 | 0.124 | 0.18 | 0.68 | 0.0007 |
|  |  | Predators | 1 | 0.0001 | 0.0001 | 0.99 | 0.0000006 |
|  |  | Site x Predators | 1 | 0.486 | 0.68 | 0.41 | 0.003 |
|  |  | Birds x Predators | 1 | 0.083 | 0.12 | 0.73 | 0.0005 |
|  |  | Site x Birds x Predators | 1 | 4.418 | 6.22 | **0.013** | 0.026 |
|  |  | Model | 7 | 17.669 | 3.56 | **0.001** |  |
|  |  | Error | 211 | 149.797 |  |  |  |
|  |  | C. Total | 218 | 167.465 |  |  | 0.076 |

Table S2. Tests of bird effects as predators on invertebrate predators and manual removals of invertebrate predators during the manipulations in each exposure and zone and by site. Variance explained is eta-squared for all factors except the total, which is Adj. R^2^.

| **Taxon** | **Exposure and Zone** | **Source** | **DF** | **SS** | **F** | **p** | **Variance explained (proportion)** | **Tukey HSD** | | |
| --- | --- | --- | --- | --- | --- | --- | --- | --- | --- | --- |
| *Nucella* spp. | Exposed Mid | Site | 3 | 12425.6 | 14.08 | **<0.0001** | 0.276 | \| YB \| A \|  \|  \| \| --- \| --- \| --- \| --- \| \| SH \| A \| B \|  \| \| FC \|  \| B \| C \| \| BB \|  \|  \| C \| | | |
|  |  | Bird effect | 1 | 1119.3 | 3.81 | 0.055 | 0.025 |  | | |
|  |  | Site x bird effect | 3 | 1957.9 | 2.22 | 0.094 | 0.044 |  | | |
|  |  | Predator removal | 1 | 5872.3 | 19.97 | **<0.0001** | 0.130 | +P > -P | | |
|  |  | Site x predator removal | 3 | 4069.3 | 4.61 | **0.0055** | 0.090 | \| YB +P \| A \|  \|  \| \| --- \| --- \| --- \| --- \| \| SH +P \| A \| B \|  \| \| YB -P \|  \| B \| C \| \| FC +P \|  \| B \| C \| \| FC -P \|  \|  \| C \| \| SH -P \|  \|  \| C \| \| BB +P \|  \|  \| C \| \| BB -P \|  \|  \| C \| | | |
|  |  | Bird effect x predator removal | 1 | 345.01 | 1.17 | 0.28 | 0.008 |  | | |
|  |  | Site x bird effect x predator removal | 3 | 392.73 | 0.45 | 0.72 | 0.009 |  | | |
|  |  | Model | 15 | 26182.2 | 5.94 | **<0.0001** | 0.582 |  | | |
|  |  | Error | 64 | 18820.5 |  |  | 0.418 |  | | |
|  |  | C. Total | 79 | 45002.7 |  |  | 0.484 |  | | |
|  | | | | | | | | | | |
| *P. ochraceus* & *Leptasterias* sp. | Exposed Mid | Site | 3 | 2038.9 | 19.39 | **<0.0001** | 0.388 | \| FC \| A \|  \|  \| \| --- \| --- \| --- \| --- \| \| BB \| A \| B \|  \| \| YB \|  \| B \|  \| \| SH \|  \|  \| C \| | | |
|  |  | Bird effect | 1 | 20.56 | 0.59 | 0.45 | 0.004 |  | | |
|  |  | Site x bird effect | 3 | 14.49 | 0.14 | 0.94 | 0.003 |  | | |
|  |  | Predator removal | 1 | 580.11 | 16.55 | **0.0001** | 0.111 | +P > -P | | |
|  |  | Site x predator removal | 3 | 303.55 | 2.89 | **0.042** | 0.058 | \| FC+P \| A \|  \|  \|  \| \| --- \| --- \| --- \| --- \| --- \| \| BB+P \| A \| B \|  \|  \| \| FC -P \|  \| B \| C \|  \| \| YB+P \|  \| B \| C \| D \| \| BB -P \|  \| B \| C \| D \| \| YB -P \|  \|  \| C \| D \| \| SH+P \|  \|  \| C \| D \| \| SH -P \|  \|  \|  \| D \| | | |
|  |  | Bird effect x predator removal | 1 | 34.74 | 0.99 | 0.32 | 0.007 |  | | |
|  |  | Site x bird effect x predator removal | 3 | 12.60 | 0.12 | 0.95 | 0.002 |  | | |
|  |  | Model | 15 | 3004.99 | 5.71 | **<0.0001** | 0.573 |  | | |
|  |  | Error | 64 | 2243.62 |  |  | 0.427 |  | | |
|  |  | C. Total | 79 | 5248.61 |  |  | 0.472 |  |  |  |
|  | | | | | | | | | | |
| *P. ochraceus* & *Leptasterias* sp. | Exposed Low | Site | 1 | 77.35 | 11.45 | **0.0017** | 0.212 | BB > SH | | |
|  |  | Bird effect | 1 | 4.50 | 0.67 | 0.42 | 0.012 |  |  |  |
|  |  | Site x bird effect | 1 | 6.60 | 0.98 | 0.33 | 0.018 |  |  |  |
|  |  | Predator removal | 1 | 33.62 | 4.98 | **0.032** | 0.092 | +P > -P | | |
|  |  | Site x predator removal | 1 | 1.09 | 0.16 | 0.69 | 0.003 |  |  |  |
|  |  | Bird effect x predator removal | 1 | 0.65 | 0.10 | 0.76 | 0.002 |  |  |  |
|  |  | Site x bird effect x predator removal | 1 | 0.11 | 0.02 | 0.9 | 0.0003 |  |  |  |
|  |  | Model | 7 | 121.75 | 2.57 | **0.029** | 0.334 |  |  |  |
|  |  | Error | 36 | 243.19 |  |  | 0.666 |  |  |  |
|  |  | C. Total | 43 | 364.94 |  |  | 0.204 |  |  |  |
